# Increased TIMP-3 expression alters the cellular secretome through dual inhibition of the metalloprotease ADAM10 and ligand-binding of the LRP-1 receptor

**DOI:** 10.1038/s41598-018-32910-4

**Published:** 2018-10-02

**Authors:** Simone D. Scilabra, Martina Pigoni, Veronica Pravatá, Tobias Schätzl, Stephan A. Müller, Linda Troeberg, Stefan F. Lichtenthaler

**Affiliations:** 1 German Center for Neurodegenerative Diseases (DZNE), Feodor-Lynen Strasse 17, 81377 Munich, Germany; 2 Neuroproteomics, School of Medicine, Klinikum rechts der Isar, Technische Universität München, 81675 Munich, Germany; 3 Kennedy Institute of Rheumatology, University of Oxford, Roosevelt Drive, Oxford, OX3 7FY UK; 4 Munich Cluster for Systems Neurology (SyNergy), Munich, Germany; 5 Institute for Advanced Study, Technische Universität München, Munich, Germany

## Abstract

The tissue inhibitor of metalloproteinases-3 (TIMP-3) is a major regulator of extracellular matrix turnover and protein shedding by inhibiting different classes of metalloproteinases, including disintegrin metalloproteinases (ADAMs). Tissue bioavailability of TIMP-3 is regulated by the endocytic receptor low-density-lipoprotein receptor-related protein-1 (LRP-1). TIMP-3 plays protective roles in disease. Thus, different approaches have been developed aiming to increase TIMP-3 bioavailability, yet overall effects of increased TIMP-3 *in vivo* have not been investigated. Herein, by using unbiased mass-spectrometry we demonstrate that TIMP-3-overexpression in HEK293 cells has a dual effect on shedding of transmembrane proteins and turnover of soluble proteins. Several membrane proteins showing reduced shedding are known as ADAM10 substrates, suggesting that exogenous TIMP-3 preferentially inhibits ADAM10 in HEK293 cells. Additionally identified shed membrane proteins may be novel ADAM10 substrate candidates. TIMP-3-overexpression also increased extracellular levels of several soluble proteins, including TIMP-1, MIF and SPARC. Levels of these proteins similarly increased upon LRP-1 inactivation, suggesting that TIMP-3 increases soluble protein levels by competing for their binding to LRP-1 and their subsequent internalization. In conclusion, our study reveals that increased levels of TIMP-3 induce substantial modifications in the cellular secretome and that TIMP-3-based therapies may potentially provoke undesired, dysregulated functions of ADAM10 and LRP-1.

## Introduction

The tissue inhibitors of metalloproteinases (TIMPs) are a family of proteins that modulate extracellular matrix (ECM) turnover by regulating the proteolytic activity of matrix metalloproteinases (MMPs)^1^. Among the four mammalian TIMPs, TIMP-3 has the broadest inhibitory profile, being able to inhibit not only MMPs, but also members of the related families of disintegrin metalloproteinases (ADAMs) and ADAMs with thrombospondin motifs (ADAMTSs). TIMP-3 is a major regulator of proteolysis *in vivo* and its genetic ablation in mouse induces a number of disease-like phenotypes, including dysregulated inflammation and signs of arthritis^2,3^. TIMP-3 is a secreted, soluble protein. ECM levels of TIMP-3 are finely regulated, and this occurs at different levels: transcriptionally, in response to growth factors or by promoter methylation^4,5^, post-transcriptionally by microRNAs^6,7^ and post-translationally by endocytosis *via* the low-density lipoprotein receptor-related protein-1 (LRP-1)^8,9^, an endocytic receptor belonging to the LDL receptor family^10^.

In humans, loss of TIMP-3 is associated with several pathological conditions. Decreased levels of TIMP-3 are present in degraded human osteoarthritis cartilage compared to normal cartilage^11^. Low expression of TIMP-3 and subsequent lack of metalloproteinase inhibition has been associated with poor prognosis in a range of human cancers^12,13^ and with the pathogenesis of inflammatory vascular disorders^14^. Conversely, increased TIMP-3 levels have been shown to be protective in diseases characterized by enhanced proteolysis, and strategies to increase its bioavailability in the tissue have been widely sought as potential therapeutics. For example, intra-articular injection of TIMP-3 inhibited cartilage breakdown in a rat model of osteoarthritis^15^, and adenovirus-mediated gene delivery of TIMP-3 reduced invasion and survival of melanoma^16^. Nevertheless, overall effects of increased TIMP-3 on cell behavior are unknown, and it is difficult to predict whether TIMP-3-based therapies may lead to side effects due to sustained high levels of the inhibitor. Indeed, recent proteomic-based studies have uncovered a large number of novel metalloproteinase substrates and, therefore, new biological roles for these enzymes, suggesting that their aberrant inhibition can lead to detrimental consequences for the tissue^17^.

In this study, we used shotgun proteomics and label free quantification (LFQ) in order to evaluate consequences in the cell secretome induced by increased TIMP-3 expression, and identified proteins and the related molecular pathways that can be altered by high levels of the inhibitor. We found that TIMP-3 had a dual effect leading to a) inhibited shedding of several transmembrane proteins, in particular substrates of ADAM10 and b) increased levels of several LRP-1 ligands, likely due to a competition for their binding to the endocytic receptor.

## Results

### TIMP-3 overexpression inhibits shedding of several ADAM10 substrates

**Figure 1:**
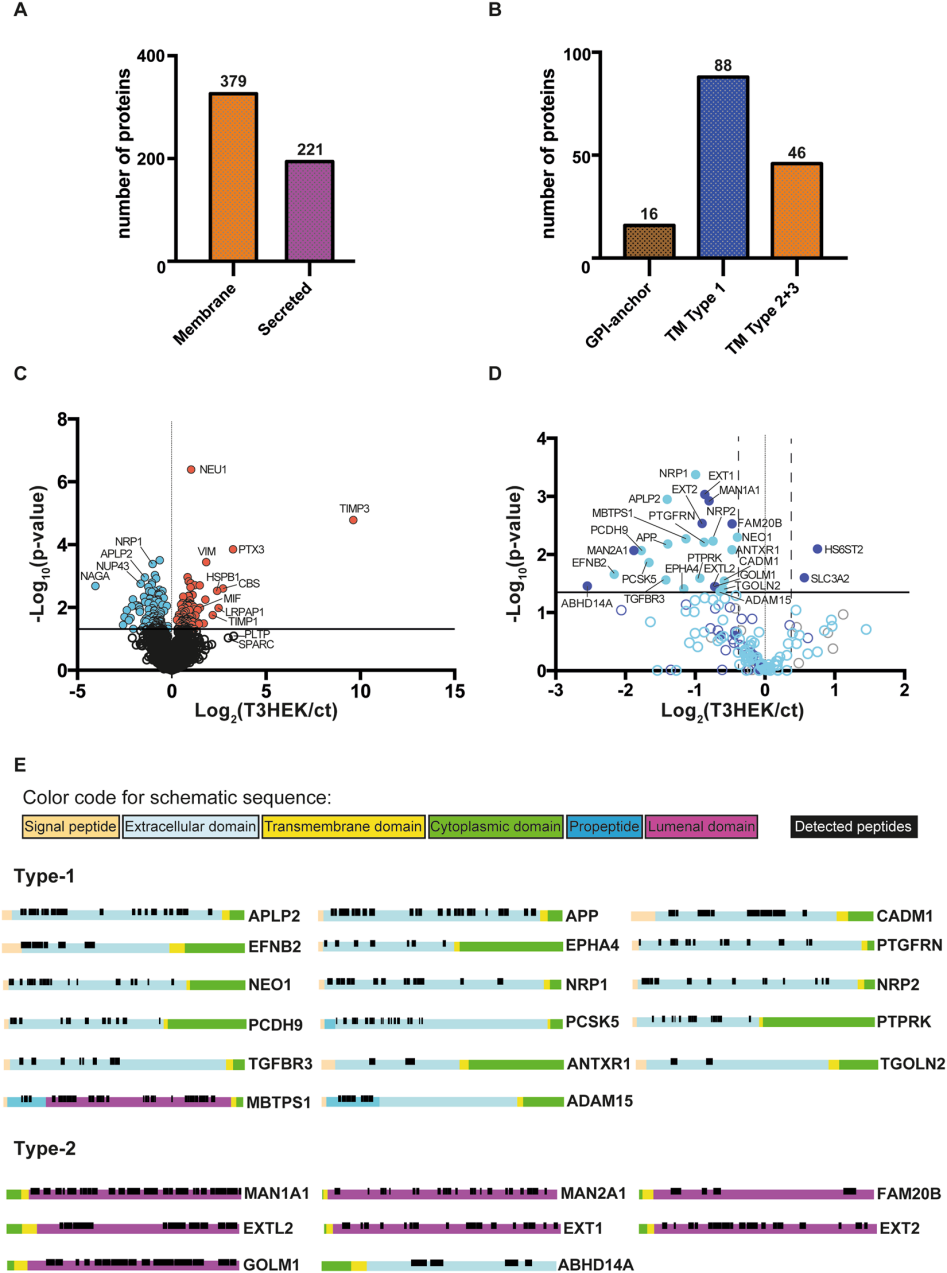
Secretome analysis of TIMP-3/HEK cells. (**A**) Number of proteins annotated as membrane or secreted proteins out of 1740 proteins in total detected in the secretome of TIMP-3/HEK cells. (**B**) Topology of single-span membrane proteins detected in the secretome of TIMP-3/HEK cells. (**C**) Volcano plot showing the −log10 of p-values versus the log2 of protein ratio between TIMP-3 overexpressing HEK293 cells (T3HEK) and control HEK293 cells (ct) of 1740 proteins (n = 6). The horizontal line indicates the –log10(p-value) of 1.3, which corresponds to a p-value of 0.05. Proteins above this line are considered significantly regulated (red dots for upregulated proteins, light blue dots for downregulated proteins). (**D**) Volcano plot showing the 88 type-1 (light blue), 46 type-2 (dark blue) and 16 GPI-anchored (grey) transmembrane proteins detected in the secretome. Filled dots represent proteins significantly regulated in the secretome of TIMP-3 overexpressing cells (p-value below 0.05 – above the horizontal solid line - difference more than 30% - vertical dashed lines), while open dots represent proteins not significantly regulated. (**E**) QARIP analysis of the transmembrane proteins that were found significantly reduced in the secretome of TIMP-3-overexpressing cells. QARIP is a webserver that matches identified peptides to the protein transmembrane topology^19^.

**Figure 2:**
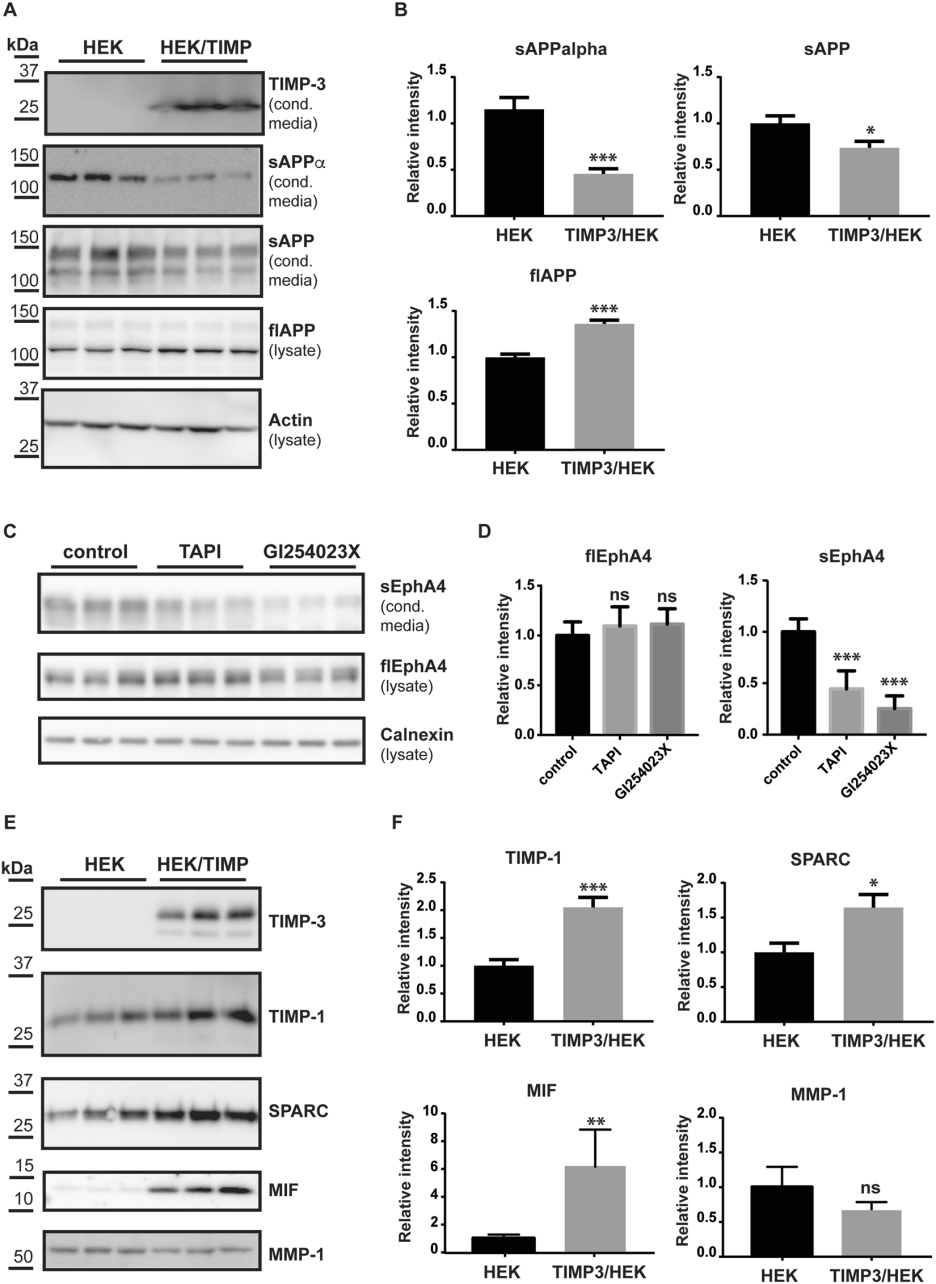
Validation of TIMP3/HEK secretome analysis by immunoblotting. (**A**,**B**) Immunoblots (**A**) and their respective quantification (**B**) showing the expression of TIMP-3, sAPP, sAPPα in the conditioned media of TIMP3/HEK and control HEK293 cells, and full length APP and actin in cell lysates (densitometric quantifications shown as mean values ± standard deviation; *p < 0.05, **p < 0.01, ***p < 0.005, Student’s t-test; 3 independent experiments are displayed out of 6 quantified, n = 6). **C-D**. Immunoblots (**C**) and their respective quantifications (**D**) showing the decrease of shed EphA4 (sEphA4) in the conditioned media of HEK cells cultured in the presence of TAPI or GI254023X (densitometric quantifications shown as mean values ± standard deviation; *p < 0.05, **p < 0.01, ***p < 0.005, Student’s t-test; 3 independent experiments are displayed out of 6 quantified, n = 6). (**E**,**F**) Immunoblots (**C**) and their respective quantification (**D**) showing the increase of TIMP-1, MIF and SPARC in the conditioned media of TIMP-3 expressing cells (densitometric quantifications shown as mean values ± standard deviation; *p < 0.05, **p < 0.01, ***p < 0.005, Student’s t-test; 3 independent experiments are displayed out of 6 quantified, n = 6). MMP-1 is shown as an example of a protein that does not increase in the conditioned media of TIMP-3/HEK293.

We used unbiased proteomics to evaluate effects of increased TIMP-3 expression on cell secretion and shedding. We analysed the conditioned media of TIMP-3 stably overexpressing HEK293 cells (TIMP3/HEK^18^) grown in the absence of serum for 48 h. Out of all proteins detected in the conditioned media of either TIMP3-overexpressing or control HEK293 cells, 221 were annotated as secreted proteins (based on Uniprot annotation) and 379 as membrane proteins (Fig. 1A). The 379 proteins annotated as membrane proteins comprise both integral and peripheral membrane proteins. Among them are 16 GPI-anchored proteins, 88 type 1 and 46 type 2 and 3 integral single-span membrane proteins (Fig. 1B, Supplemental Table 1). Label-free quantification (LFQ) analysis confirmed that low levels of endogenous TIMP-3 were detected in the conditioned medium of control HEK293 cells, with a clear increase in the conditioned media of the TIMP-3 stably transfected cells (Fig. 1C, Table 1. As a result of the increased TIMP3 expression, numerous additional proteins besides TIMP-3 showed altered levels in the secretome (Fig. 1C). Proteins were arbitrarily considered as hits when the p-value of their change was below 0.05 (measured with a two-sided Student t-test without correction for multiple hypothesis testing) and when their levels changed by at least 30%. This yielded 210 regulated proteins, of which 114 exhibited reduced levels and 96 exhibited increased levels, including TIMP-3. Figure 1D displays selectively the membrane proteins out of all identified proteins. Among the membrane proteins with reduced levels were 17 proteins annotated as type-1 and 8 annotated as type-2 membrane proteins, suggesting that the increased TIMP3 levels may have reduced the proteolytic release of their ectodomains. (Fig. 1D). To test this possibility, the ‘quantitative analysis of regulated intramembrane proteolysis’ (QARIP) webserver was used, which allows mapping identified peptides to the protein transmembrane topology^19^. QARIP analysis of TIMP3/HEK secretome revealed that the identified peptides of these transmembrane proteins match only with their ectodomain, but not transmembrane or intracellular domains, in agreement with an inhibition of their metalloproteinase-mediated shedding in the presence of increased TIMP-3 (Fig. 1E). Seven out of the 17 type-1 membrane proteins, including amyloid precursor protein-like 2 (APLP2)^20,21^, amyloid precursor protein (APP)^22–24^, cell adhesion molecule 1 (CADM1)^25^, Ephrin-B2 (EFNB2)^26^, neogenin 1 (NEO1)^27^, neuropilin-1 (NRP1)^28^ and receptor-type tyrosine-protein phosphatase kappa (PTPRK)^27^, have previously been identified as ADAM10 substrates, indicating that TIMP3 predominantly blocks activity of ADAM10 in this cellular system. To validate the mass spectrometry LFQ results, we used immunoblotting as an orthogonal method to analyse the shedding of one of the reduced membrane proteins, the known ADAM10 substrate APP^22,24,29^. Indeed, levels of total soluble APP (sAPP) and sAPPα (which is specifically generated by ADAM10 cleavage^22^) in the conditioned media were lower in the TIMP-3 expressing cells (Fig. 2A,B), indicating decreased ADAM10-dependent shedding of APP in agreement with the mass spectrometry data and previous reports^30^. As a result of the reduced shedding, full length APP was slightly more abundant in TIMP-3/HEK cell lysates. As a control, immunoblotting clearly detected TIMP-3 in the conditioned media of TIMP3/HEK cells, while it was almost undetectable in the conditioned media of HEK293 cells (Fig. 2A,E).

**Table 1:** Changes in protein secretion in TIMP3 expressing cells.

Protein name	Gene	ID	Ratio	p-value	pept
**Selected soluble proteins increased in the conditioned medium of TIMP3 expressing cells**					
**Metalloproteinase inhibitor 3**	**TIMP3**	**P35625**	**793**.**91**	**1**.**65E-05**	**17**
Pentraxin-related protein PTX3	PTX3	P26022	9.55	1.41E-04	18
Metalloproteinase inhibitor 1	TIMP-1	P01033	4.51	1.77E-02	13
Macrophage migration inhibitory factor	MIF	P14174	2.82	1.02E-02	6
**Selected soluble proteins decreased in the conditioned medium of TIMP3 expressing cells**					
Galectin-3-binding protein	LGALS3BP	Q08380	0.18	1,99E-02	34
Laminin subunit alpha-4	LAMA4	LAMA4	0.21	2.55E-02	43
Plasma alpha-L-fucosidase	FUCA2	Q9BTY2	0.24	4.85E-02	22
**Selected membrane proteins showing reduced shedding in TIMP3 expressing cells**					
Ephrin-B2	EFNB2	P52799	0.22	2.18E-02	5
Protocadherin-9	PCDH9	Q9HC56	0.29	8.61E-03	14
Proprotein convertase subtilisin/kexin type-5	PCSK5	Q92824	0.31	1.37E-02	19
Transforming growth factor beta receptor type 3	TGFBR3	Q03167	0.36	2.73E-02	8
Amyloid-like protein 2	APLP2	Q06481	0.37	1.11E-03	22
Amyloid beta A4 protein	APP	P05067	0.37	6.66E-03	29
Ephrin type-A receptor 4	EPHA4	P54764	0.44	3.89E-02	7
Neuropilin-1	NRP1	O14786	0.49	4.16E-04	15
Receptor-type tyrosine-protein phosphatase kappa	PTPRK	Q15262	0.51	2.58E-02	13
Prostaglandin F2 receptor negative regulator	PTGFRN	Q9P2B2	0.54	6.15E-03	13
Cell adhesion molecule 1	CADM1	Q9BY67	0.65	2.86E-02	8
Anthrax toxin receptor 1	ANTXR1	Q9H6X2	0.71	8.25E-03	3
Neogenin	NEO1	Q92859	0.75	5.00E-03	20

*ID*: Uniprot accession number of the protein.

*Ratio*: mean ratio of label-free quantification intensities between HEK/TIMP-3 and control HEK293 cells (n = 6).

*p-value*: for six biological replicates.

*pept*: number of identified unique peptides of the protein group.

Besides known ADAM10 substrates, the ectodomains of 18 other transmembrane proteins were also found to be less abundant in the secretome of TIMP-3 expressing cells, including transforming growth factor beta receptor type 3 (TGFBR3), which has been identified as an MMP-14 substrate^31^, ephrin type-A receptor 4 (EphA4) and neuropilin-2, shed by an as yet unidentified metalloproteinase^32,33^ (Table 1, Fig. 1D, Supplemental Table 1). In addition, the ectodomains of protocadherin-9 (PCDH9), prostaglandin F2 receptor negative regulator (PTGFRN), proprotein convertase subtilisin/kexin type-5 (PCSK5), anthrax toxin receptor 1 (ANTXR1), Trans-Golgi network integral membrane protein 2 (TGOLN2) and alpha/beta hydrolase domain-containing protein 14A (ABHD14A) were less abundant in the conditioned media of TIMP-3 expressing cells, indicating that these proteins can also undergo metalloproteinase-dependent shedding (Fig. 1D, Table 1, Supplemental Table 1), potentially by ADAM10. In agreement with this hypothesis, we confirmed by immunoblotting that shedding of EphA4 was reduced in the presence of the metalloproteinase inhibitor TAPI and by the ADAM10 specific inhibitor GI254023X^34,35^ (Fig. 2C,D). Nevertheless, we can’t exclude that proteins belonging to this group can be shed by another TIMP-3 dependent metalloproteinase or regulated by TIMP-3 through an undefined mechanism. All identified peptides of ADAM15 matched with the pro-domain of the enzyme (Fig. 1E) that, similarly to other ADAMs, is cleaved in the Golgi by furin^36^. This suggests that TIMP-3 can reduce the pro-domain removal of ADAM15, and its subsequent secretion in the extracellular environment. The last subgroup of these 17 membrane proteins with reduced levels comprised 8 further transmembrane proteins, 1 type-1 and 7 type-2, which are the membrane-bound transcription factor site-1 protease (MBTPS1), mannosyl-oligosaccharide 1,2-alpha-mannosidase IA (MAN1A1), Alpha-mannosidase 2 (MAN2A1), glycosaminoglycan xylosylkinase (FAM20B), exostosin-like 2 (EXTL2), exostosin-1 (EXT1), exostosin-2 (EXT2), golgi membrane protein 1 (GOLM1), mainly located in the endoplasmic reticulum or Golgi apparatus (Fig. 1D, Supplemental Table 1). As the QARIP analysis had mapped the identified peptides within the luminal domain of these proteins, it is likely that they have undergone metalloproteinase-dependent shedding in the Golgi, where metalloproteases, such as ADAM10, can be active^37^, and that the luminal domains were subsequently released into the extracellular milieu (Fig. 1E). Similarly to the ectodomain of transmembrane proteins, a number of ECM proteins, including laminins, was found less abundant in the conditioned media of TIMP-3-overexpressing cells (Table 1, Supplemental Table 1).

Collectively, these data indicate that overexpression of TIMP-3 in HEK293 cells reduces shedding of a diverse group of transmembrane proteins, majorly by inhibiting the activity of ADAM10.

### Overexpression of TIMP-3 increases levels of LRP-1 ligands

**Figure 3:**
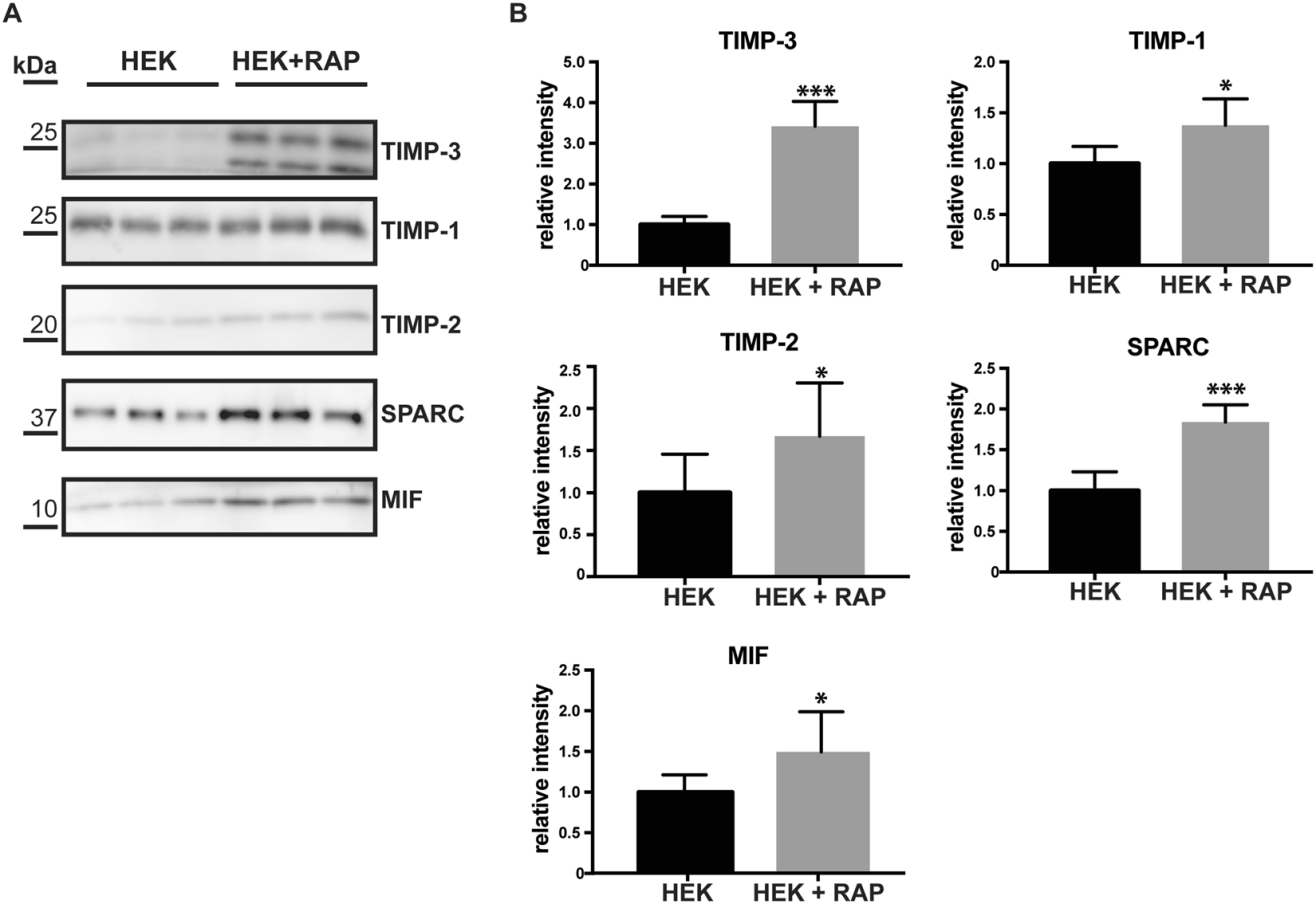
RAP increases levels of specific proteins. (**A**,**B**) Immunoblots and their respective quantification showing levels of specific proteins (TIMP-3, TIMP-1, TIMP-2, SPARC, MIF) in the conditioned media of HEK293 cells in the presence or absence of RAP (densitometric quantifications shown as mean values ± standard deviation; *p < 0.05, **p < 0.01, ***p < 0.005, Student’s t-test; 3 independent experiments are displayed out of 6–8 quantified, n = 6–8).

**Figure 4:**
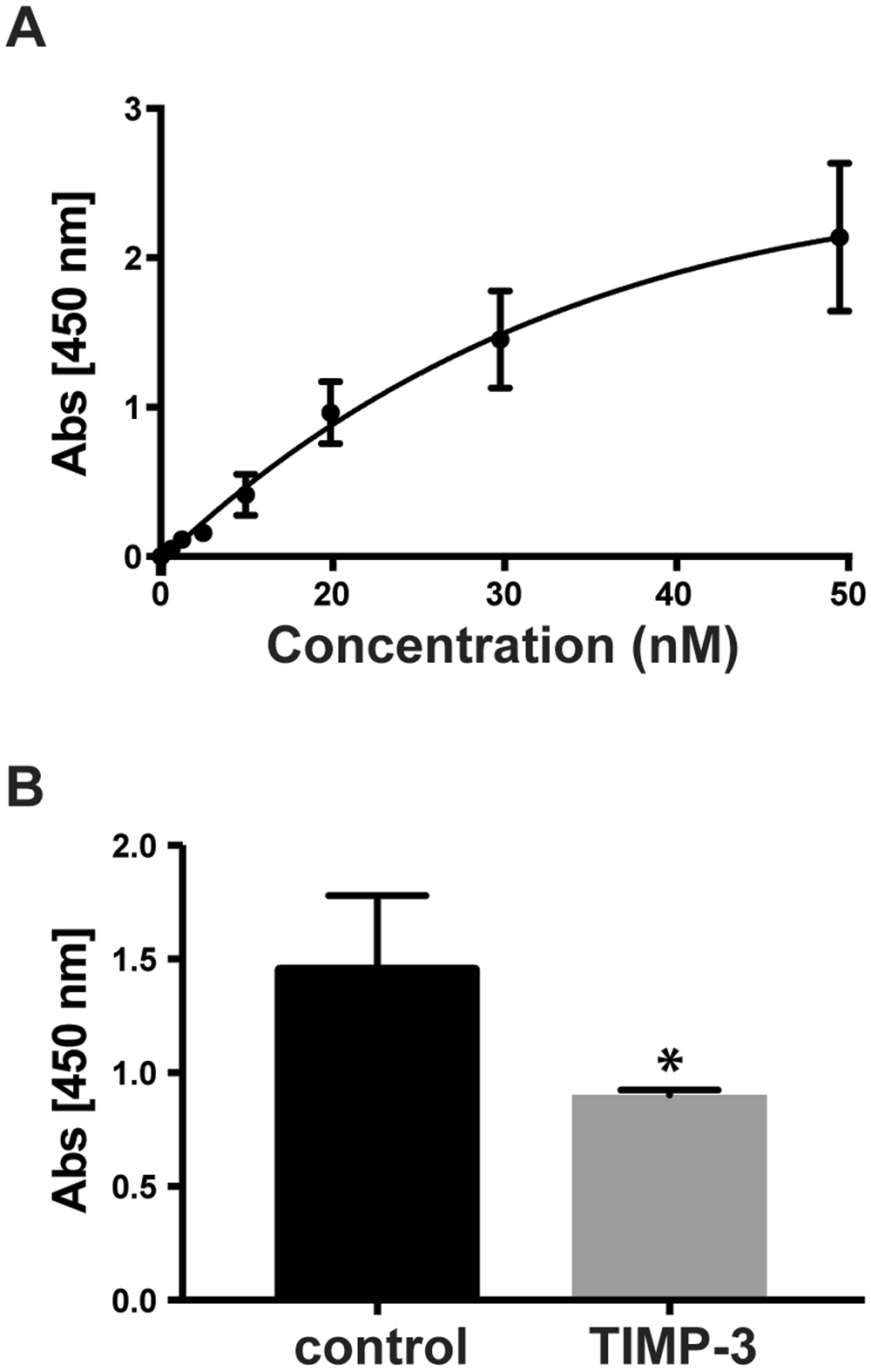
TIMP-3 competes with TIMP-1 for its binding to LRP-1. (**A**) Full-length LRP1 was coated onto microtiter plates and binding of FLAG-tagged TIMP-1 (0–48 nM) was measured using an M2 anti-FLAG antibody (n = 3). (**B**) Similarly, binding of 24 nM FLAG-tagged TIMP-1 to LRP-1 was measured in the absence (control) or presence of 2 nM TIMP-3.

The secretome analysis showed that expression of TIMP-3 not only reduced shedding of several membrane proteins, but also induced an increase in levels of several soluble proteins, such as the TIMP-3-homolog TIMP-1, macrophage migration inhibitory factor 1 (MIF1), osteonectin (SPARC), tissue factor pathway inhibitor (TFPI) and pentraxin-related protein (PTX3) (Fig. 1C, Table 1, Supplemental Table 1). Selected proteins – TIMP-1, SPARC and MIF – were further analyzed by immunoblots, where the increased levels seen in the mass spectrometric measurement were confirmed (Fig. 2E,F). This suggests an involvement of TIMP-3 in the regulation of these proteins. Mechanistically, this may occur through their shared binding to LRP-1. TIMP-3 binds LRP-1, and extracellular levels of TIMP-3 are known to be regulated by LRP-1^8^, an endocytic receptor that binds to and internalizes over fifty proteins via four ligand-binding domains (I-IV)^10^. Different LRP-1 ligands bind to different ligand-binding domains, but also to different binding sites within the same ligand-binding domain^38,39^. Similarly to TIMP-3, TIMP-1^40^ and TFPI^41^ – identified above – are known LRP-1 ligands. Thus, we hypothesized that overexpression of TIMP-3, and its subsequent engagement with this endocytic receptor, could increase levels of other ligands that compete with TIMP-3 for their binding to LRP-1. To test this possibility, we analysed whether similar protein level increases as seen with TIMP-3 expression are observed when binding of LRP-1 to its ligands is blocked. To this aim, we used the receptor-associated protein (RAP), a known inhibitor of LRP-1, which binds to all four ligand-binding domains with the highest affinity among LRP-1 ligands, thus blocking the uptake of all known ligands, and not only of TIMP-3^10^. In support of our hypothesis, we found in immunoblots that TIMP-1, MIF and SPARC, which were increased in TIMP-3-overexpressing cells, also accumulated in the conditioned media of cells treated with RAP (Fig. 3A,B). In addition, to confirm the competition hypothesis, we analysed whether TIMP-3 could compete with TIMP-1 for its binding to LRP-1 *in vitro*. In an ELISA assay, TIMP-1 bound to LRP-1 with a K_D,App_ of 16 nM (Fig. 4A), in agreement with previous reports^40^. The addition of 2 nM TIMP-3 (a concentration similar to that of its binding K_D_ for the receptor^38^) was sufficient to reduce the amount of TIMP-1 that binds to LRP-1 by about 40% (Fig. 4B). Levels of TIMP-2, another known LRP-1 ligand^42^, accumulated as expected in the conditioned media in the presence of RAP (Fig. 3A,B), but were not increased in TIMP-3/HEK (Fig. 1C). This suggests that TIMP-2 may bind to a separate site from that of TIMP-3, so that RAP, but not TIMP-3-overexpression affects its turnover and therefore its extracellular levels.

**Figure 5:**
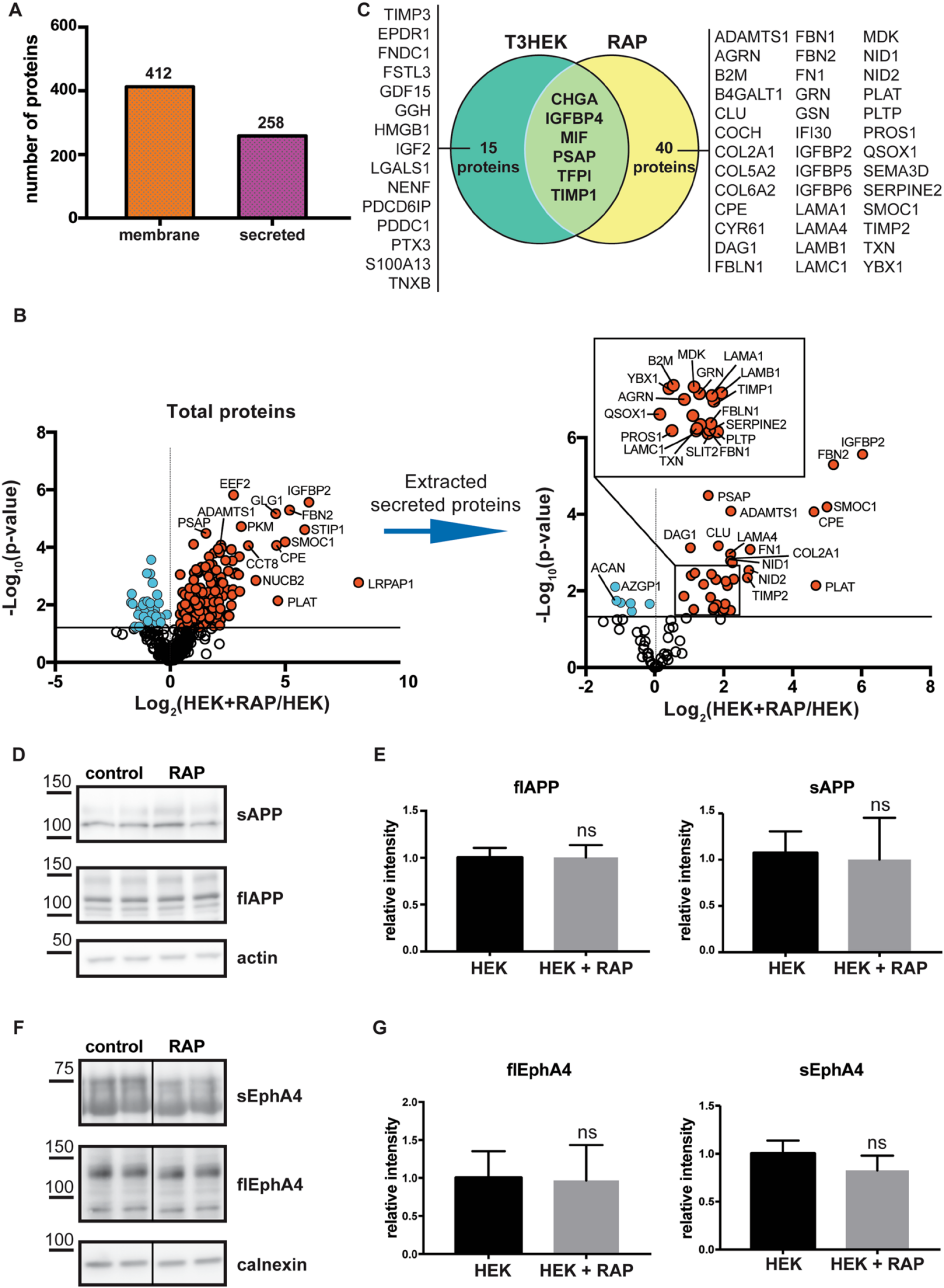
Secretome analysis of RAP-treated HEK293 cells. (**A**) Topology of all the proteins detected in the secretome of RAP-treated HEK293 cells. (**B**) Volcano plot showing the −log10 of p-values versus the log2 of protein ratio between HEK293 cells in the presence and absence of RAP *(left)*. RAP itself was also identified and is shown in the plot under the gene name ‘LRPAP1’. From this analysis, a volcano plot showing only secreted proteins was extracted *(right)*. The horizontal solid line indicates the –log10(p-value) of 1.3 (corresponding to a p-value of 0.05). Proteins above this line are considered significantly regulated (red dots for upregulated proteins, light blue dots for downregulated proteins, (n = 6). (**C**) Venn diagram showing secreted proteins that are upregulated in either TIMP-3/HEK or RAP-treated HEK293 secretomes (p-values < 0.05, and proteins found only in TIMP-3/HEK or RAP-treated secretomes, but not in the control). In the intersection between the two diagrams are listed proteins upregulated in both secretomes. (**D**–**G**) Immunoblots (**D**,**F**) and their respective quantifications (**E**–**G**) showing that addition of 500 nM RAP to HEK293 cells does not alter shedding of APP (**D**,**E**) and EphA4 (**F**,**G**, displayed bands were run on the same blot but not directly next to each other) (densitometric quantifications shown as mean values ± standard deviation; *p < 0.05, **p < 0.01, ***p < 0.005, Student’s t-test; 2 independent experiments are displayed out of 6 quantified, n = 6).

Besides the LRP-1 ligands TIMP-1, MIF, SPARC and TFPI, additional soluble proteins were upregulated in TIMP-3 expressing cells (Fig. 1C). To determine which of them may be additional potential LRP-1 ligands, we analysed the conditioned media of RAP-treated HEK293 cells by using a similar proteomic approach as in Fig. 1 and compared the results with the secretome of TIMP-3/HEK cells. The secretome analysis of RAP-treated cells identified 412 proteins annotated as membrane proteins and 258 proteins annotated as secreted ones (Fig. 5A,B). Together with RAP (identified by the gene name LRPAP1 in the volcano plot), levels of 34 secreted proteins were significantly (i.e. proteins whose levels changed by at least 30% with a p-value of their change below 0.05) increased in the presence of RAP (Fig. 5B). As expected, TIMP-1, MIF and TFPI, which were increased in the secretome of TIMP-3-overexpressing cells, were also found increased in the secretome of RAP-treated cells. MIF and TFPI were only found in the secretome of RAP-treated but not control cells and are therefore not shown in the volcano plot in Fig. 5C, (see also Supplemental Table 1). Similarly, IGFBP4 and CHGA were only found in the secretome of TIMP-3/HEK and RAP-treated HEK cells, but not in their respective controls. Additionally, PSAP was upregulated in the secretome of both TIMP-3/HEK and RAP-treated cells (Fig. 5C), suggesting that all three proteins were upregulated in the TIMP-3/HEK secretome and that this is because they are new LRP-1 ligands. In addition, RAP treatment increased levels of a higher number of secreted proteins compared to TIMP-3-overexpression, presumably because they have binding sites in LRP-1 that are different from the TIMP-3 binding site. Among these, many are already established LRP-1 ligands, including TIMP-2^42^, the tissue-type plasminogen activator (PLAT)^43^ and midkine (MDK)^44^ (Fig. 5B,C, Table 2). Other secreted proteins which were upregulated in the presence of RAP, including the insulin-like growth factor-binding protein 2 (IGFBP-2), ADAMTS-1 and the glia-derived nexin (SERPINE2), have high probability to be novel LRP-1 ligands, as they belong to families of proteins that are known to interact with the receptor. Indeed, other members of the IGFBP family of proteins^45^, several serpins^46^ and the other two aggrecanases ADAMTS-4 and -5^47,48^ have been identified as LRP-1 ligands. Conversely, only 6 secreted proteins and the ectodomains of 2 transmembrane proteins (DSG1 and PCDH7) were reduced upon RAP treatment, while the majority of shed ectodomains were not altered, indicating that RAP had no major effects on protein shedding (Fig. 5B and Supplemental Table 1). In agreement, immunoblotting confirmed that RAP did not alter the shedding of APP and EphA4 (Fig. 5D–G).

**Table 2:** SECRETED proteins increased in the conditioned medium of RAP-TREATED HEK293 cells.

Protein name	Gene	ID	Ratio	p-value	pept
**Alpha-2-macroglobulin receptor-associated protein**	**LRPAP1**	**P30533**	**229**.**36**	**1**.**67E-03**	**63**
Insulin-like growth factor-binding protein 2	IGFBP2	P18065	65,40	2.71E-06	16
Fibrillin-2	FBN2	P35556	36.40	5.07E-06	50
SPARC-related modular calcium-binding protein 1	SMOC1	Q9H4F8	31.97	6.43E-05	18
Tissue-type plasminogen activator	PLAT	P00750	25.58	7.18–03	21
Carboxypeptidase E	CPE	P16870	24.61	8.57E-05	19
Fibronectin	FN1	P02751	6,81	8.23E-04	63
Nidogen-2	NID2	Q14112	4.60	1.64E-03	53
Metalloproteinase inhibitor 2	TIMP2	P16035	6.46	4.50E-03	10
Laminin subunit beta-1	LAMB1	P07942	4.89	4.91E-03	60
Collagen alpha-1	COL2A1	P02458	4.77	1.83E-03	10
A disintegrin and metalloproteinase with thrombospondin motifs 1	ADAMTS1	Q9UHI8	4.62	8.25E-05	28
Nidogen-1	NID1	P14543	4.60	1.64E-03	56
Laminin subunit alpha-4	LAMA4	Q16363	4.60	1.09E-03	39
Phospholipid transfer protein	PLTP	P55058	4.59	3.21E-02	11
Metalloproteinase inhibitor 1	TIMP1	P01033	4.30	7.29E-03	8
Fibulin-1	FBLN1	P23142	4.12	2.18E-02	29
Glia-derived nexin	SERPINE2	P07093	4.02	2.98E-02	13

*ID*: Uniprot accession number of the protein.

*Ratio*: mean ratio of label-free quantification intensities between RAP-treated and control HEK293 cells (n = 6).

*p-value*: for six biological replicates.

*pept*: number of identified peptides of the protein group.

In conclusion, our study suggests that levels of a number of secreted proteins, including TIMP-1, are increased in TIMP-3-overexpressing cells as a consequence of their competition with TIMP-3 for the binding to LRP-1. In addition to them, we identified several proteins accumulating in the conditioned media upon LRP-1 inactivation, that are likely to be novel LRP-1 ligands.

## Discussion

TIMP-3 is an endogenous inhibitor of metalloproteinases, and as such, an essential regulator of ECM turnover^1^. TIMP-3 has proved beneficial in diseases characterized by enhanced proteolysis, including arthritis and cancer^13,15^; thus, approaches to increase its bioavailability in the tissue have been developed for the therapy of such diseases^16,38,49^. However, given that TIMP-3 has numerous targets and binding partners, a therapeutic increase of TIMP-3 levels may lead to mechanism-based side effects. In order to predict molecular pathways that can be affected by high doses of TIMP-3, we took advantage of a quantitative, high-resolution mass spectrometric workflow to investigate changes in the cell secretome upon overexpression of the inhibitor. By using this method, we identified two major classes of proteins whose release is altered in the presence of high TIMP-3 levels: transmembrane proteins that undergo metalloproteinase-mediated shedding, in particular ADAM10 substrates, and LRP-1 ligands that may compete with TIMP-3 for the binding to the receptor.

First, we found that the ectodomain of several transmembrane type-1 proteins detected in the conditioned media were decreased in the presence of TIMP-3, suggesting inhibition of their ectodomain shedding. A large fraction of these proteins are established substrates of ADAM10, including APP^37,50^. In addition, TIMP-3 overexpression led to a decrease of different transmembrane protein ectodomains whose sheddase has not been identified yet, including protocadherin-9, ephrin type-A receptor 4, prostaglandin F2 receptor negative regulator and anthrax toxin receptor 1, indicating them as novel substrates of a metalloproteinase, potentially of ADAM10. In addition to being the physiological α-secretase of the Alzheimer APP protein^22^, ADAM10 controls several biological processes by shedding various substrates^37,50^. Its ablation in mouse is embryonically lethal, as a result of the loss of cleavage and signaling of the Notch receptor, a well-characterized ADAM10 substrate^51^. Thus, we speculate that sustained TIMP-3 expression, and the consequent persistent inhibition of ADAM10, as well as the inhibition of other metalloproteinases, can have detrimental effects *in vivo*. In support of this hypothesis, transgenic mice overexpressing TIMP-3 in chondrocytes display abnormalities in bone development, including decreased osteoblast differentiation and reduction in growth plate length and bone mass^52,53^. Such a phenotype may be due to dysregulation of Notch signaling, which plays a crucial role in maintaining bone homeostasis by regulating proliferation and differentiation of both osteoblasts and osteoclasts^54^. Furthermore, we found that overexpression of TIMP-3 led to a decrease in abundance of a number of soluble proteins in the secretome, including galectin-3-binding protein, plasma alpha-L-fucosidase and several laminin subunits. Laminins are heterotrimeric proteins (containing an alpha, a beta and a gamma-chain) that connect cells to the ECM by binding cell surface receptors, such as integrins, and ECM components^55^. Several laminin subunits, including laminin alpha-4, alpha-1, alpha-5, beta-1 and gamma-1 were decreased in the conditioned media in the presence of high levels of TIMP-3. This indicates that overexpression of TIMP-3 may stabilize laminin receptors on the cell surface, thereby decreasing soluble levels of these proteins in the extracellular milieu.

Second, overexpression of TIMP-3 promoted accumulation of a number of secreted proteins that are known to be LRP-1 ligands, including TIMP-1, another member of the TIMP family^40^. We hypothesized that the accumulation of TIMP-1 in the conditioned media of TIMP-3-overexpressing cells can be due to its competition with TIMP-3 for binding to the LRP-1 receptor. Thus, TIMP-3-overexpression may trigger a positive feedback loop that enhances the bioavailability of both TIMP-1 and TIMP-3. Similarly to TIMP-3, TIMP-1 is able to inhibit ADAM10^56^, thus, increased levels of both TIMPs upon TIMP-3 overexpression may function additively to inhibit MMP-mediated ECM proteolysis and the activity of ADAM10. In contrast to TIMP-3 that binds to LRP-1 within the N-terminal half of the ligand binding domain II^38^, RAP interacts with all four ligand binding domains, thus inhibiting the uptake of all known ligands of LRP-1. In agreement, RAP increased levels of a higher number of proteins in the secretome than was seen with TIMP-3 overexpression. Among these, RAP increased levels of proteinases, including ADAMTS-1 and PLAT, and proteinase inhibitors, such as TIMPs. Previous studies showed that, despite increasing both proteinases and proteinase inhibitors, the overall effect of LRP-1 inactivation on ECM turnover is promoting its catabolism. Indeed, LRP-1 inactivation by RAP induces aggrecan degradation in explants of porcine articular cartilage^48^, and its genetic ablation in fibroblasts increases turnover of fibronectin and collagen^57^. We found that levels of the tissue-type plasminogen activator (PLAT) were more than 25-fold higher in the presence of RAP compared to controls. PLAT is a serine protease that cleaves plasminogen into active plasmin, which in turn promotes ECM degradation^58,59^. In addition to plasminogen, PLAT can activate the urokinase-type plasminogen activator and several MMPs, including MMP-9^60–62^. It is interesting to speculate that such high levels of PLAT accumulating in the conditioned medium as a consequence of LRP-1 inactivation can trigger a proteinase activation cascade that would exceed the inhibitory potential of TIMPs and serpins, thus promoting ECM breakdown. In agreement, we found that RAP increased levels of a number of ECM proteins in the secretome, including nidogens, laminins, collagens and fibronectin, in addition to LRP-1 ligands. The increase of these ECM proteins may be due to enhanced ECM catabolism, which renders fragments of these proteins more available in the extracellular milieu.

In conclusion, TIMP-3 is considered a valuable target for the therapy of conditions associated with enhanced proteolysis, including arthritis and cancer. Different therapeutical approaches have been developed to increase the bioavailability of TIMP-3 in the tissue, including injections of recombinant TIMP-3 or adenovirus-mediated gene delivery^15,16^. Our data suggest that such approaches may affect dramatically cell behaviour, by inhibiting shedding of several proteins at the cell surface, in addition to influencing the turnover of ECM proteins. This must be taken into a consideration when developing therapies based on TIMP-3, as too high levels of the inhibitor can have harmful consequences on tissue homeostasis.

## Methods

### Secretome analysis of TIMP-3-expressing cells

TIMP-3 stably-transfected HEK293 cells (TIMP-3/HEK) were generated as previously described^18^, and maintained in DMEM with 10% FCS, 100 U/ml penicillin and 100 U/ml streptomycin, 800 μg/mL hygromycin B at 37 °C in 5% CO_2_. TIMP-3/HEK or HEK293 cells were cultured in 10 cm dishes until confluence and then incubated in serum-free DMEM. After 48 h, supernatants were harvested and centrifuged to remove cell debris. Protein concentration in supernatants was measured using the colorimetric 660 nm assay (Thermo Fisher Scientific, US). A protein amount of 30 μg per sample was subjected to proteolytic digestion using the filter assisted sample preparation (FASP) protocol with 10 kDa Vivacon spin filters (Sartorius, Germany)^63^. Proteins were digested with 0.5 μg LysC (Promega) over-night and subsequently with 0.5 μg trypsin (Promega) for 4 h at 37 °C. Peptides were desalted by stop-and-go extraction (STAGE) on reverse phase C18^64^, and eluted using 40 μl of 60% acetonitrile in 0.1% formic acid. The volume was reduced in a SpeedVac and the peptides were resuspended in 0.1% formic acid for LC-MS/MS analysis. To achieve high sensitivity, a nanoLC-MS/MS setup was used that included a nanoLC system (EASY-nLC 1000, Proxeon – part of Thermo Scientific, US) with an in-house packed C18 column (30 cm × 75 μm ID, ReproSil-Pur 120 C18-AQ, 1.9 μm, Dr. Maisch GmbH, Germany) coupled online via a nanospray flex ion source equipped with a PRSO-V1 column oven (Sonation, Germany) to a Q-Exactive mass spectrometer (Thermo Scientific, US). 1 μg of peptides were separated using a binary gradient of water and acetonitrile containing 0.1% formic acid at 50 °C column temperature, using the settings for this system shown in Table 3.

**Table 3:** LC gradient for the separation of tryptic peptides for LC-MS/MS analysis.

Time [min]	Duration [min]	Flow [nL/min]	acetonitrile [%]
0	—	50	2
5	5	250	5
185	180	250	25
230	45	250	35
250	20	250	60
255	5	250	95

### Proteomic data analysis

The data was analyzed by the software Maxquant (maxquant.org, Max-Planck Institute Munich) version 1.5.2.6. The MS data was searched against a reviewed canonical fasta database of Homo sapiens from UniProt (download: December 12^th^ 2014, 16685 entries). Trypsin was defined as protease. Two missed cleavages were allowed for the database search. The option first search was used to recalibrate the peptide masses within a window of 20 ppm. For the main search peptide and peptide fragment mass tolerances were set to 4.5 and 20 ppm, respectively. Carbamidomethylation of cysteine was defined as a static modification. Acetylation of the protein N-terminus as well as oxidation of methionine set as variable modifications. Label free quantification (LFQ) of proteins required at least two ratio counts of unique peptides. Only unique peptides were used for quantification. The LFQ values were log2 transformed and a two-sided Student’s t-test was used to evaluate proteins statistically significantly regulated between TIMP-3-overexpressing HEK293 and control cells. A p-value less than 0.05 was set as the significance threshold.

### Validation of regulated proteins by immunoblotting

A number of selected proteins that were detected as regulated in the analysis were further validated by immunoblotting (TIMP-3, TIMP-1, SPARC, MIF, APP). Supernatants from TIMP-3/HEK or HEK293 cells were loaded onto an acrylamide gel for electrophoresis. After electrophoretic separation, proteins were blotted onto a PVDF membrane using the Trans-Blot Turbo transfer system (Biorad) and detected by the following antibodies: anti-TIMP-3 (AB6000, Millipore), anti-TIMP-1 (generated as previously described^65^), anti-TIMP-2 (generated as previously described^66^), anti-SPARC (number 5031, Thermo Fisher), anti-MIF (clone FL-115, Santa Cruz) anti-APP (clone 22C11, Millipore), anti-MMP-1 (clone 2A7.2, Millipore) anti-actin (A5316, Sigma Aldrich).

Bands corresponding to each protein were quantified by using Multi Gauge software (Fujifilm) and normalized to the mean of the original non-normalized control values (HEK293 cells). A two-sided Student’s t-test was used to evaluate proteins statistically significantly regulated, with a p-value less than 0.05 that was set as the significance threshold.

### Validation of EphA4 as an ADAM10 substrate

HEK293 cells were treated with TAPI (Sigma-Aldrich SML0739, final concentration: 50 μM), GI254023X (Sigma-Aldrich, SML0789, final concentration: 5 μM) or DMSO for 24 h in serum-free DMEM. Then, conditioned media were collected and concentrated by TCA-precipitation. Cells were collected with STET lysis buffer (50 mM Tris, pH 7,5, 150 mM NaCl, 2 mM EDTA, 1% Triton), containing protease inhibitor cocktail (1:500, Sigma, P-8340). Conditioned media and cell lysates were loaded onto an acrylamide gel for electrophoresis and blotted onto a PVDF membrane. Proteins were detected with an anti-EphA4 antibody (number 610471, BD Biosciences) and anti-calnexin (number ADI-SPA-860, Enzo Life Sciences, Germany).

### ELISA to analyse binding of TIMP-1 to LRP-1

Recombinant human C-terminally FLAG-tagged TIMP-1 was generated with a modification of the method previously described for purifying TIMP-3^18^. Briefly, TIMP-1 was expressed using a pCEP4-based expression vector (Invitrogen, UK) constructed by the PCR method. Human embryonic kidney HEK293 cells were transfected with the expression plasmid by using Lipofectamine 2000 (Thermo Fisher Scientific). 48 h after transfection, cells were incubated in serum-free DMEM for 4 days. Then, conditioned medium was collected, centrifuged to remove cell debris and applied to a column of anti-FLAG M2-agarose (0.5 ml, Sigma-Aldrich). The column was washed with TNC, and bound protein eluted with 200 μg/ml FLAG peptide (Sigma-Aldrich, UK) in TNC buffer. To remove the FLAG peptide, the eluate was applied to a Vivaspin column with a 10 kDa membrane cut-off (Sartorius, Germany). The identity of the protein was confirmed by immunoblotting, and its active concentration was measured by titration against a known concentration of MMP-1 catalytic domain.

2 nM human LRP1 (Biomac, Germany) in TNC (50 mM Tris/HCl, 150 mM NaCl, 10 mM CaCl_2_, pH 7.4) was coated overnight at 4 °C onto microtiter plates. Wells were blocked with 3% BSA in TNC (1 h; 37 °C) and washed in TNC containing 0.05% (v/v) Brij-35 after this and each subsequent step. Wells were then incubated with various concentrations of FLAG-tagged TIMP-1 (0–48 nM) either alone or in the presence of 2 nM TIMP-3 (R&D Systems) in blocking solution for 3 h at room temperature. Bound TIMP-1 was detected using anti-FLAG M2 mouse monoclonal antibody (Sigma-Aldrich) and then with an anti-mouse secondary antibody coupled to horseradish peroxidase (Promega). Hydrolysis of tetramethylbenzidine substrate (slow kinetic form, Sigma-Aldrich) was measured at 450 nm using a Tecan infinite m200 pro absorbance microplate reader (Tecan, Switzerland). Results were analysed by Graphpad Prism software, which was used to generate the binding curve (using the one-site saturation binding function) and extrapolate the K_D,App_.

### Secretome analysis of RAP-treated HEK293 cells

The receptor-associated protein RAP was purified as previously described^48^. Briefly, recombinant human His-tagged RAP was expressed in Escherichia coli BL21(DE3) using a pET3a-based expression vector (Novagen/EMD Biosciences), and purified from the soluble fraction of the cell lysate by using a nickel-nitrilotriacetic acid Superflow resin (Qiagen). After washing the column with 20 mM HEPES (pH 7.5), 1 M NaCl, and 50 mM imidazole, bound protein was eluted in 20 mM HEPES (pH 7.5), 150 M NaCl, and 500 mM imidazole. HEK293 cells were cultured in 6-well plates until confluence and then incubated in serum-free DMEM with or with out 500 nM RAP for 24 h. Supernatants were harvested and analysed by LC-MS/MS using the same method used to analyse supernatants of TIMP-3/HEK cells. Briefly, supernatants were harvested and centrifuged to remove cell debris, prior to being concentrated in 10 kDa Vivacon spin filters and subjected to FASP. Proteolytic peptides were desalted using STAGE tips. The purified peptides were dried by vacuum centrifugation and resuspended in 10 μL 0.1% formic acid and analyzed by LC-MS/MS.

Some of the proteins that were significantly regulated in the conditioned media of RAP-treated HEK293 cells (TIMP-3, TIMP-1, TIMP-2, MIF and SPARC, primary antibodies described above), together with proteins whose shedding was not affected by RAP (APP and EphA4), were further validated by Western blotting. Cells were grown to confluence in 6-well plates, and then incubated for 24 h in serum-free DMEM, with or without RAP. After collection, supernatants were concentrated by TCA-precipitation and loaded onto an acrylamide gel for electrophoresis and Western blotting. Bands corresponding to each protein were quantified and analysed for their statistically significant regulation as described above. All test were done on an exploratory 2-sided 5% significance level.

## Electronic supplementary material

Uncut immunoblots

Dataset 1
