# Supplementary material for: Increased TIMP-3 expression alters the cellular secretome through dual inhibition of the metalloprotease ADAM10 and ligand-binding of the LRP-1 receptor
Source: Sci Rep. 2018 Oct 2;8:14697. doi: 10.1038/s41598-018-32910-4 (PMC6168507; doi:10.1038/s41598-018-32910-4)
Supplement: Supplementary file 1 — Uncut immunoblots [file 41598_2018_32910_MOESM1_ESM.pdf]

Increased TIMP-3 expression alters the cellular secretome through dual inhibition of the metalloprotease ADAM10 and ligand-binding of the LRP-1 receptor

Simone D. Scilabra, Martina Pigoni, Veronica Pravatá, Tobias Schätzl, Stephan A. Müller, Linda Troeberg, Stefan F. Lichtenthaler

Supplementary Information

Figure 2

TIMP3

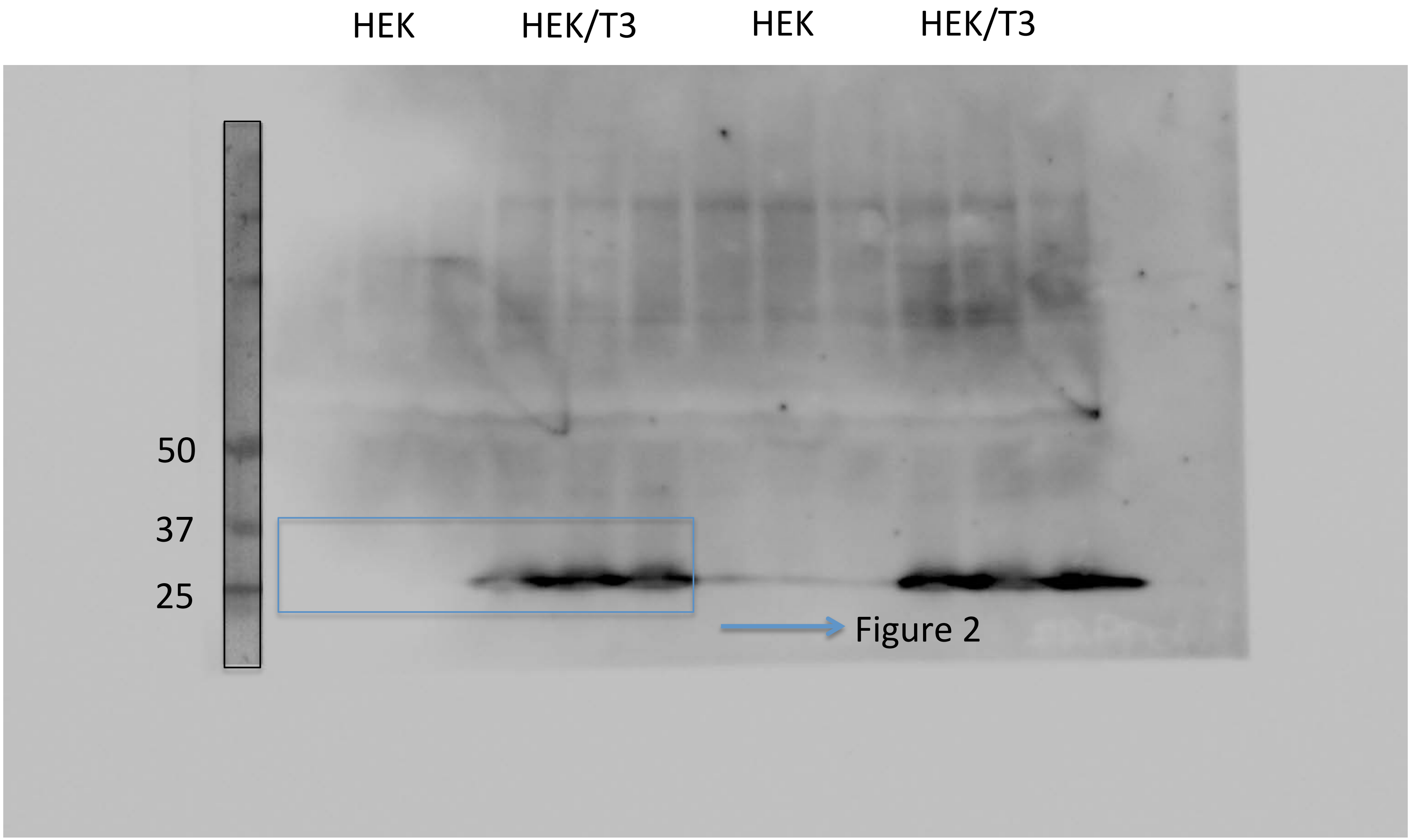

sAPPalpha

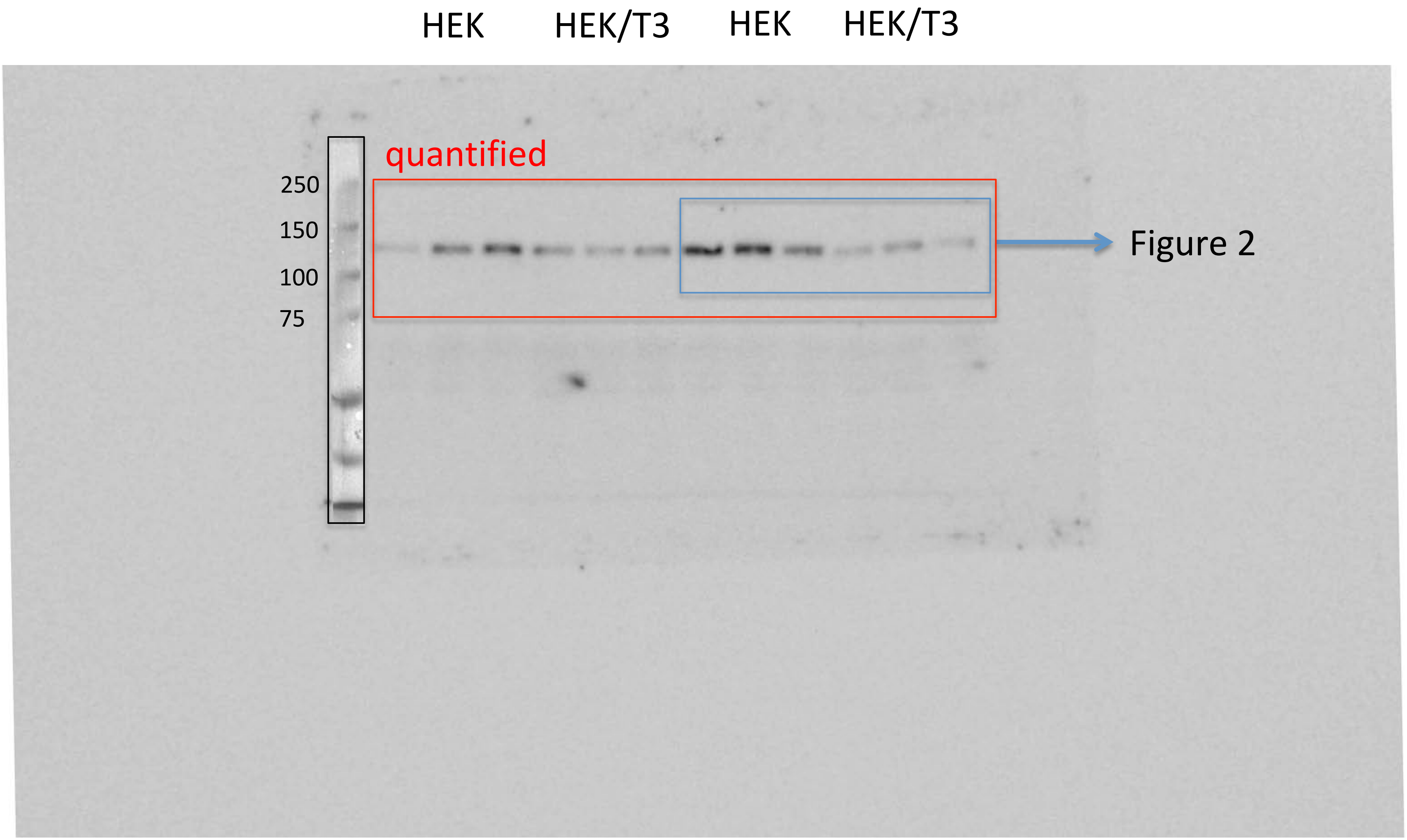

Blue square: bands in the MS. Red square: bands quantified for statistics

sAPP

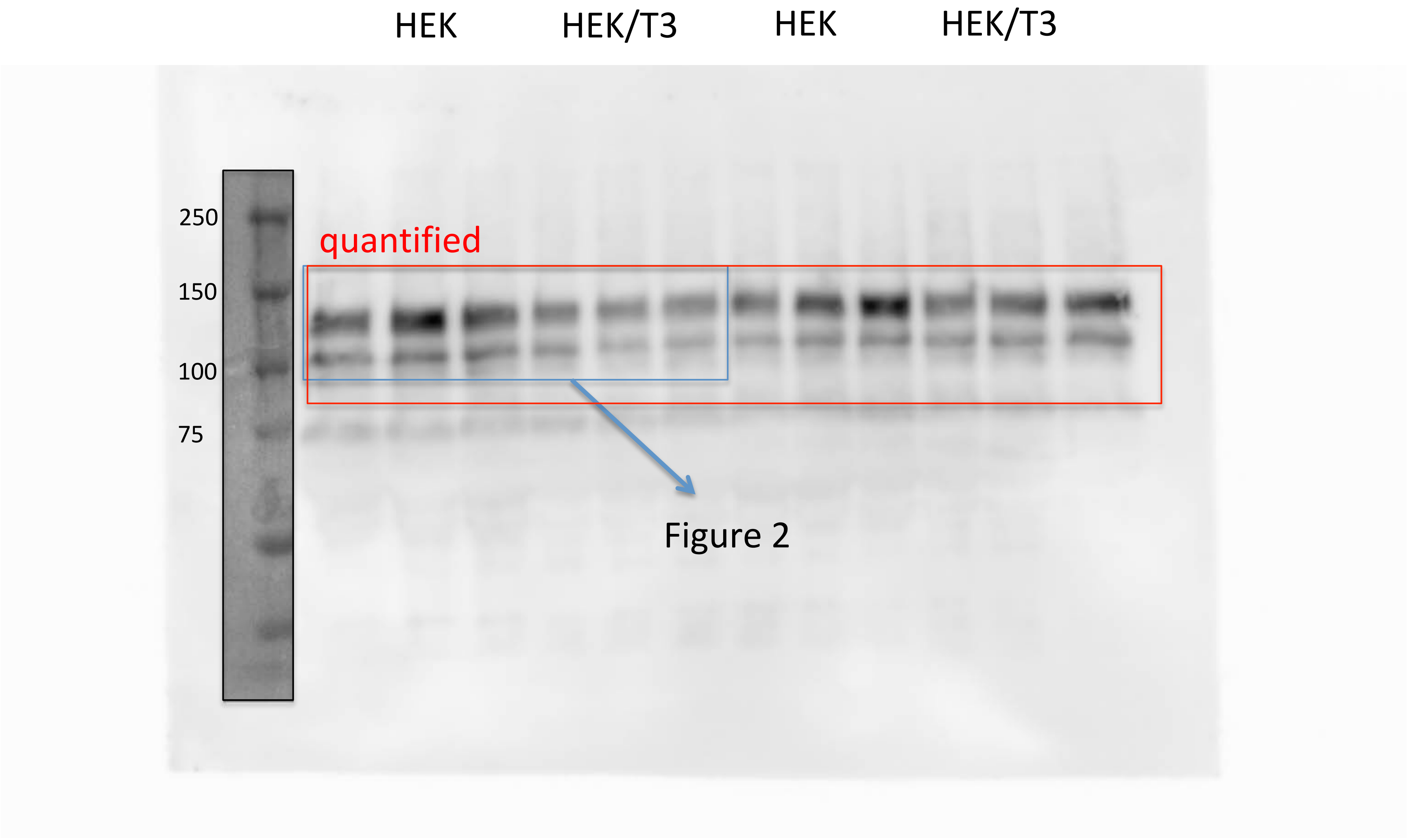

Blue square: bands in the MS. Red square: bands quantified for statistics

APPfl-22C11

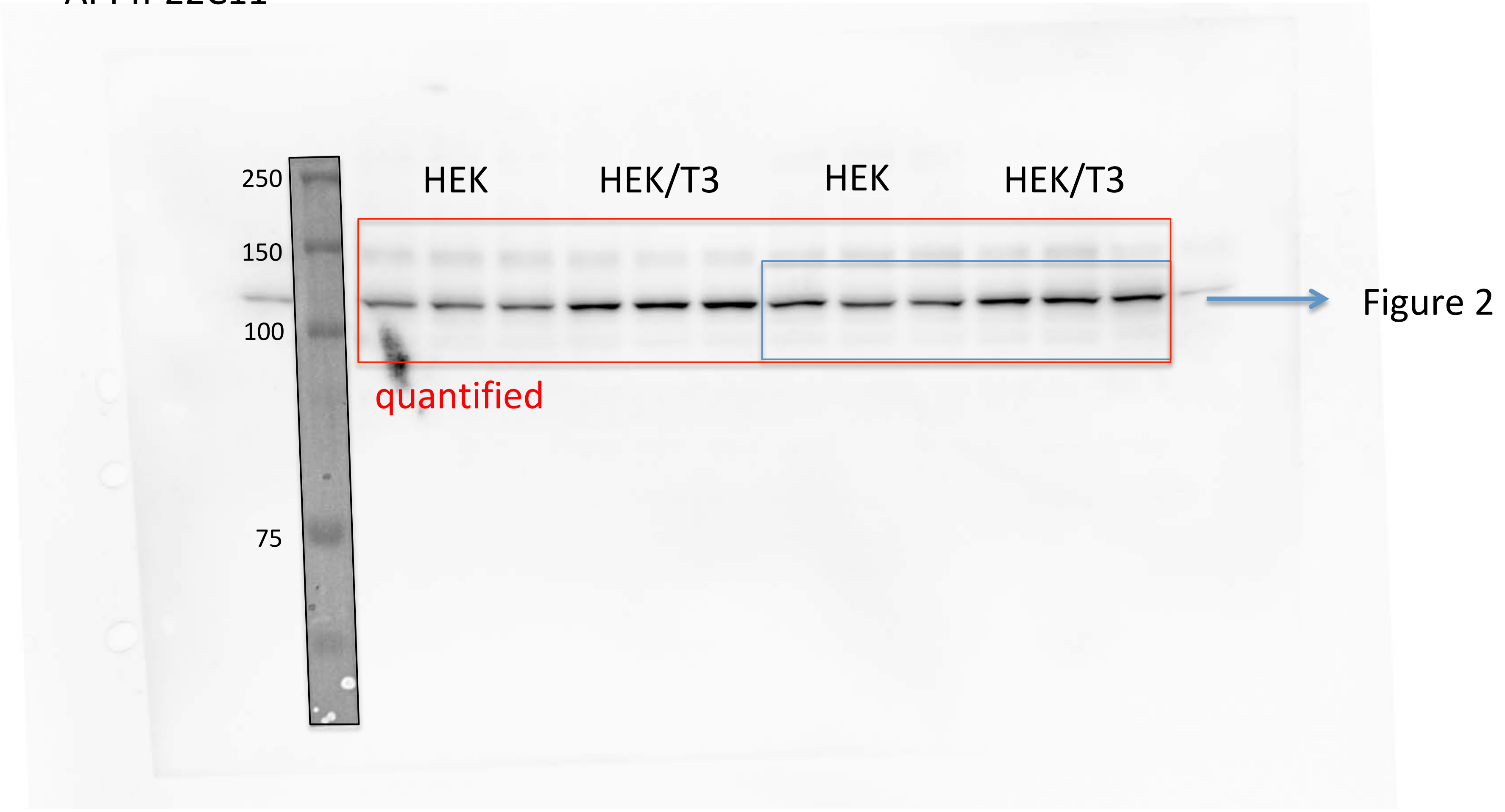

Blue square: bands in the MS. Red square: bands quantified for statistics

actin

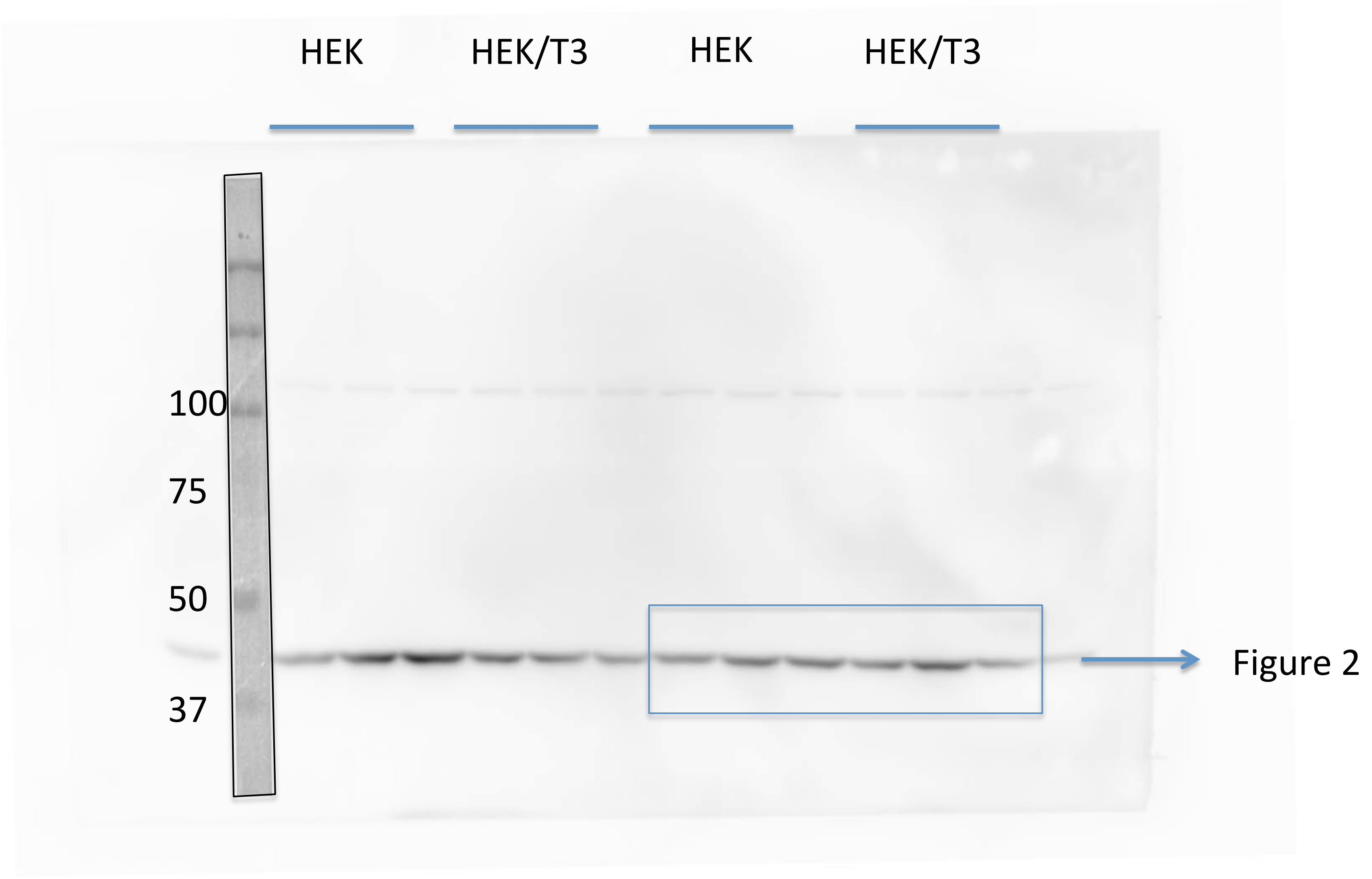

TIMP3

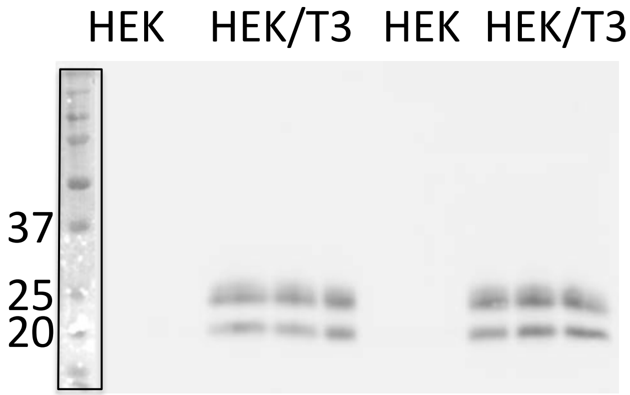

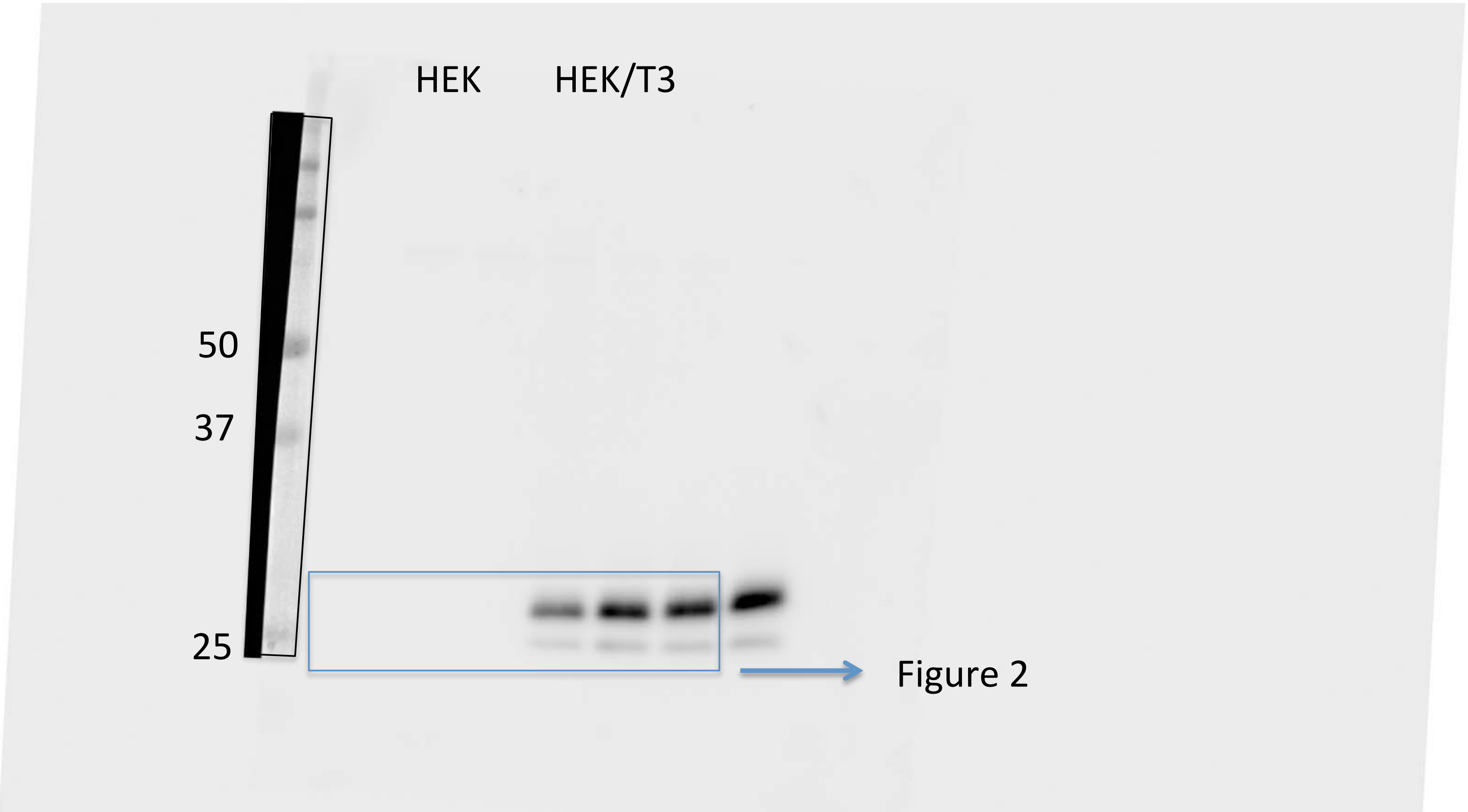

MIF

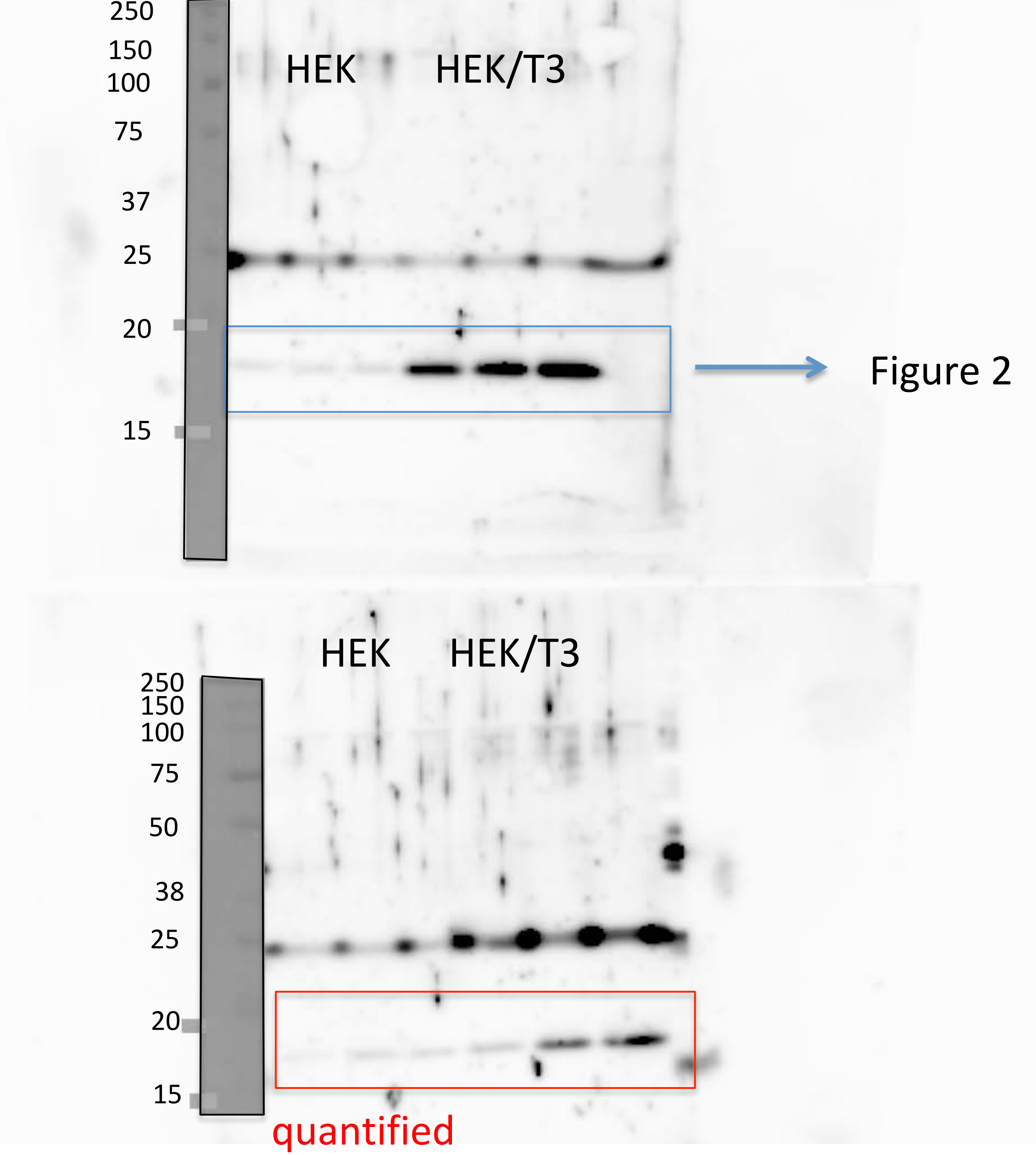

Blue square: bands in the MS. Red square + Blue square: bands quantified for statistics

TIMP1

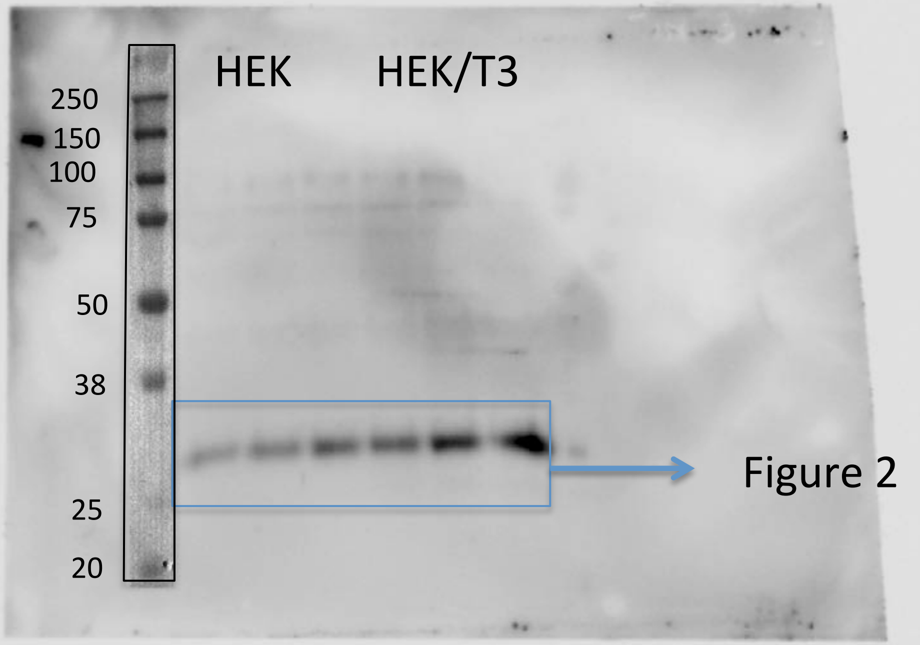

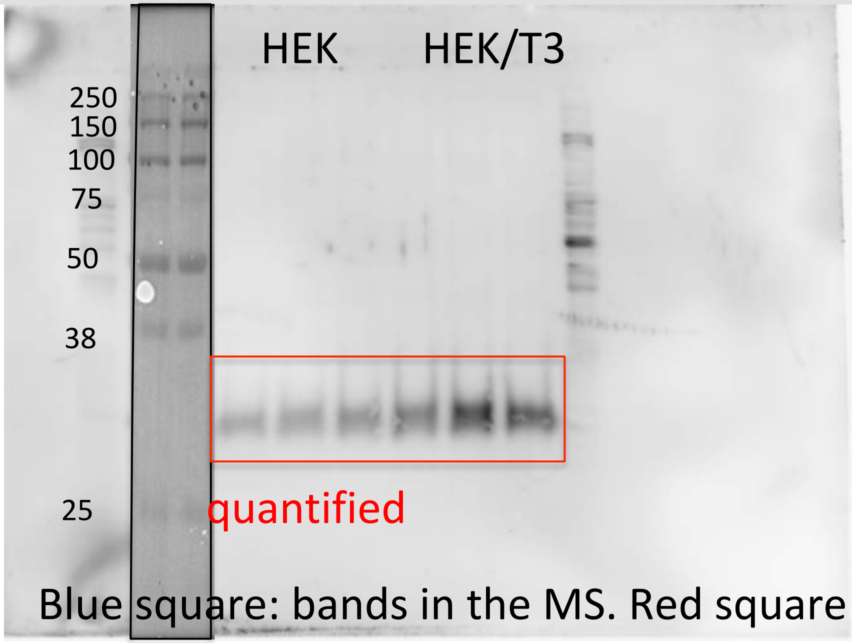

Blue square: bands in the MS. Red square + Blue square: bands quantified for statistics

MMP1

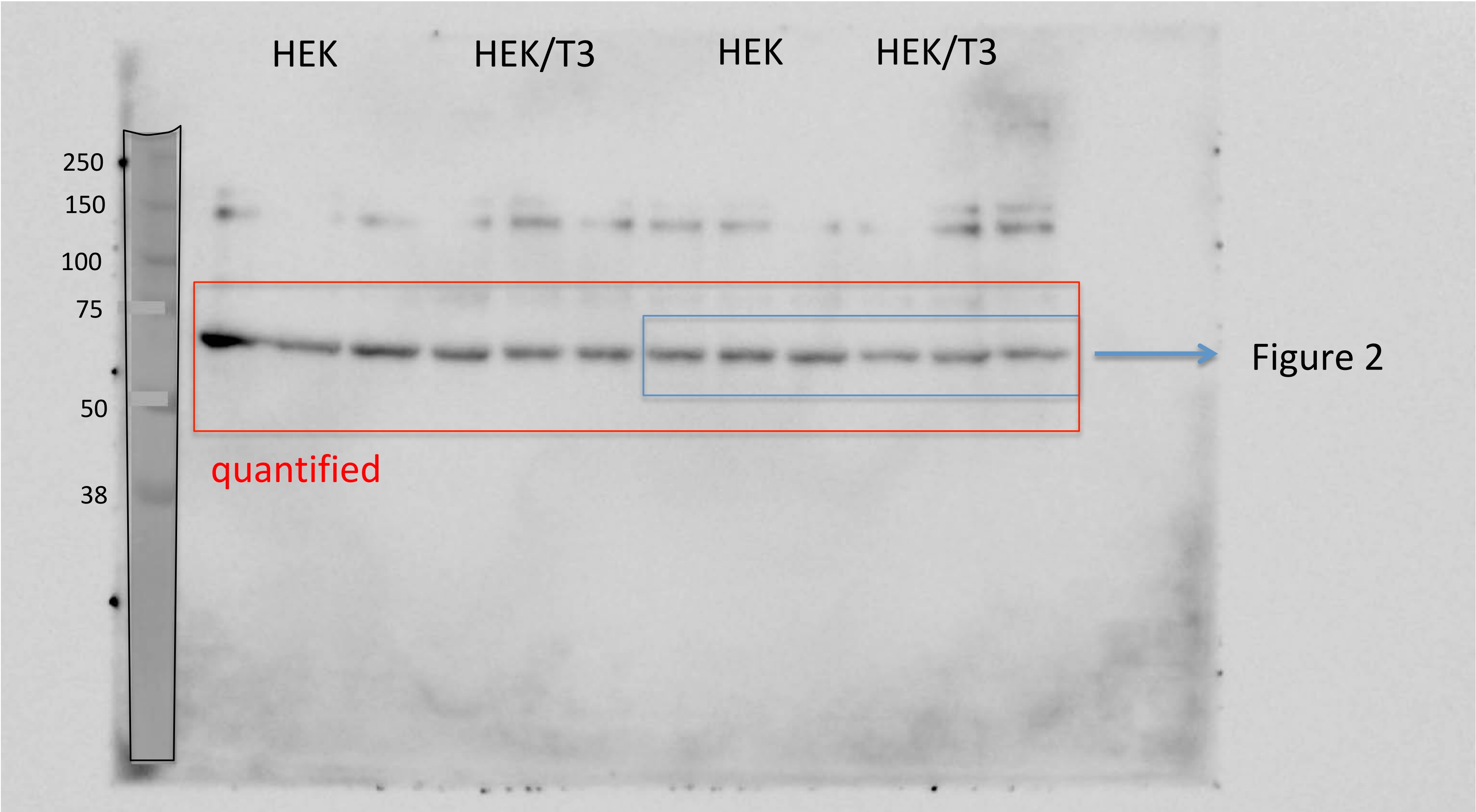

Blue square: bands in the MS. Red square: bands quantified for statistics

SPARC

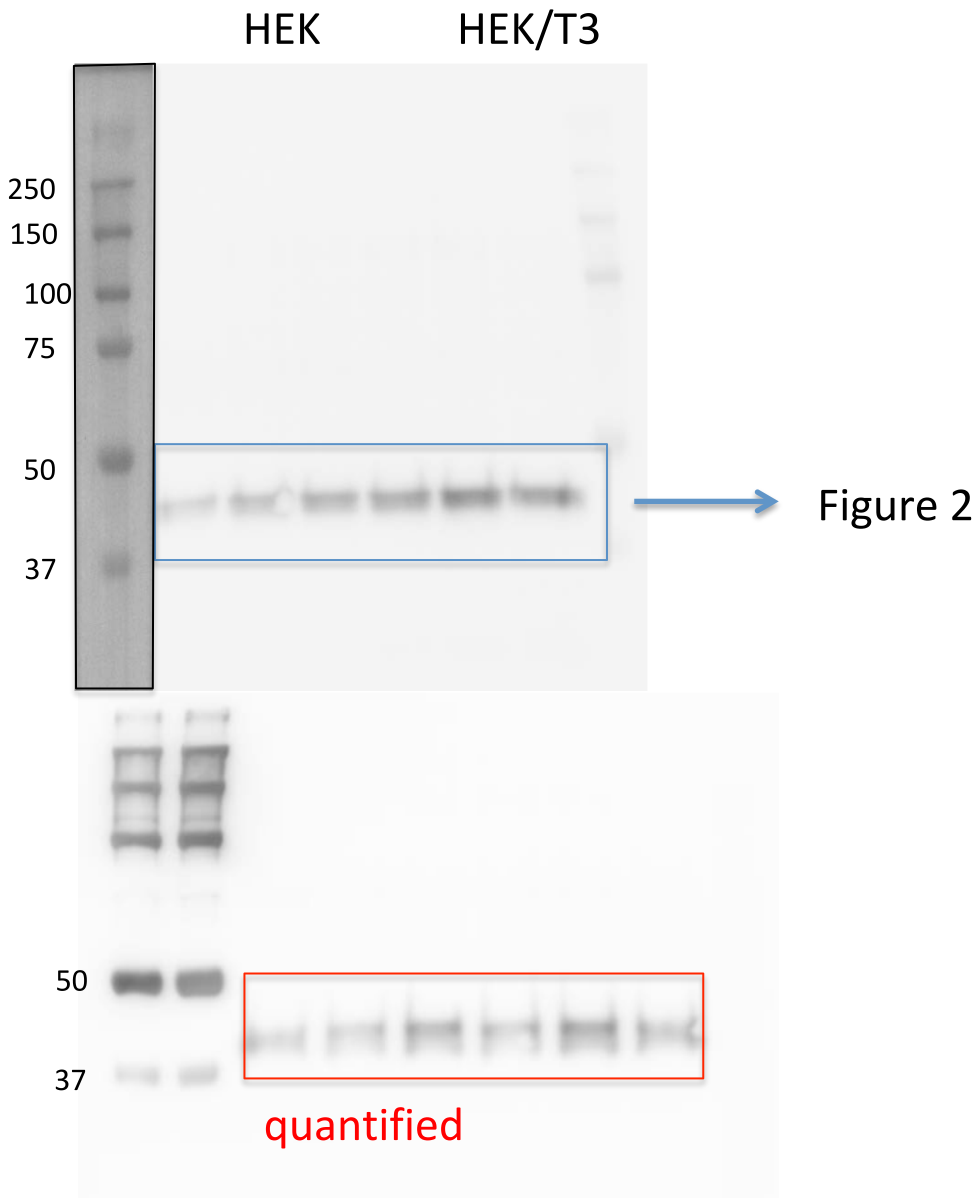

Blue square: bands in the MS. Red square + Blue square: bands quantified for statistics

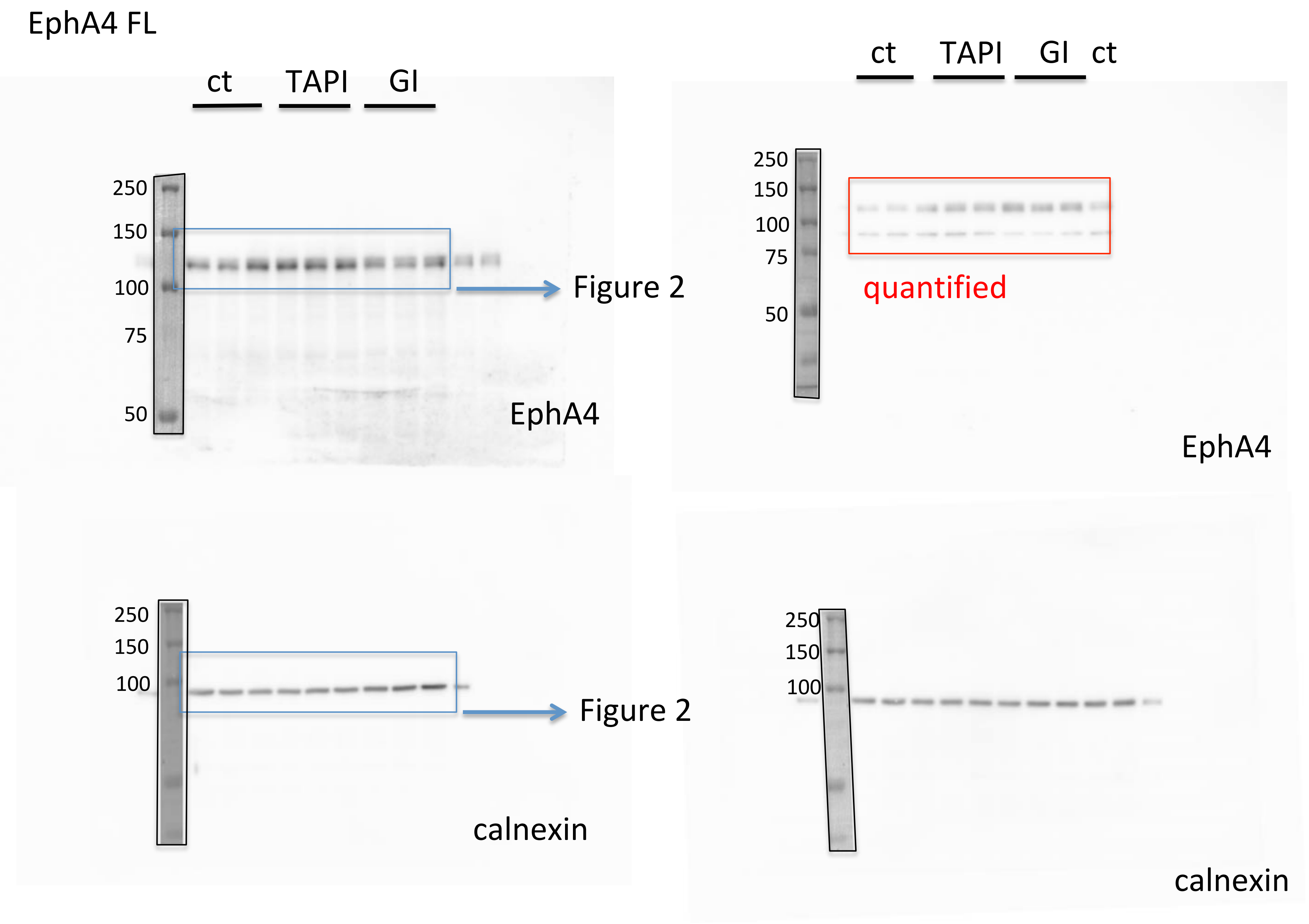

Blue square: bands in the MS. Red square + Blue square: bands quantified for statistics

sEphA4

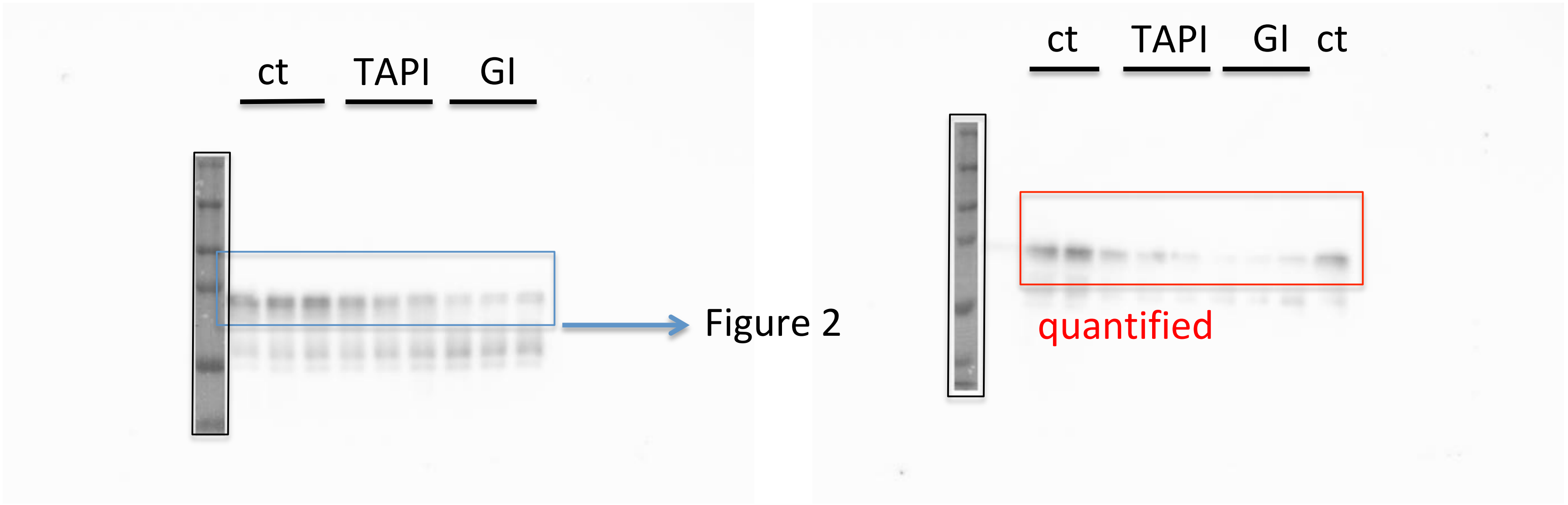

Blue square: bands in the MS. Red square + Blue square: bands quantified for statistics

Figure 3

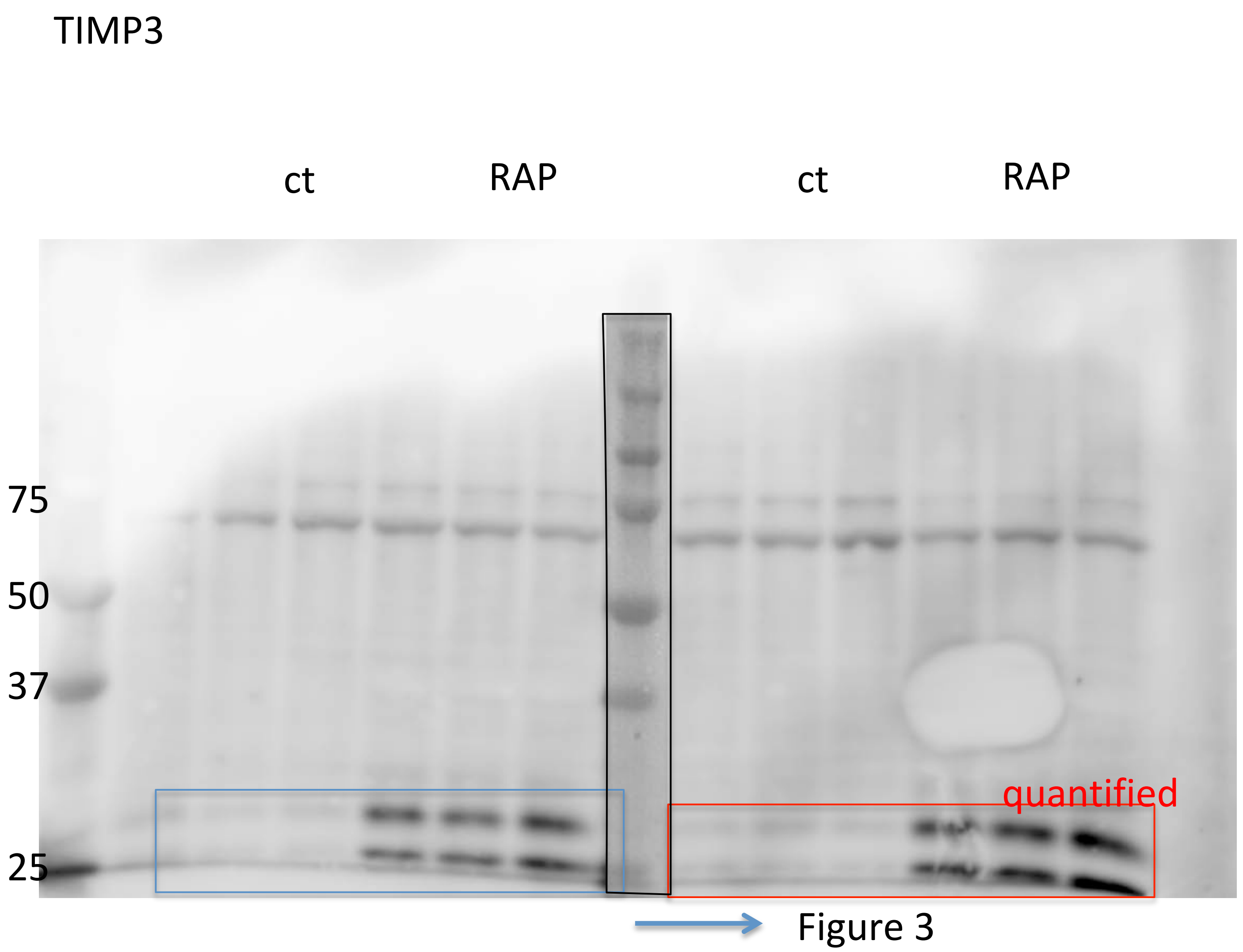

Blue square: bands in the MS. Red square + Blue square: bands quantified for statistics

TIMP1

ct RAP

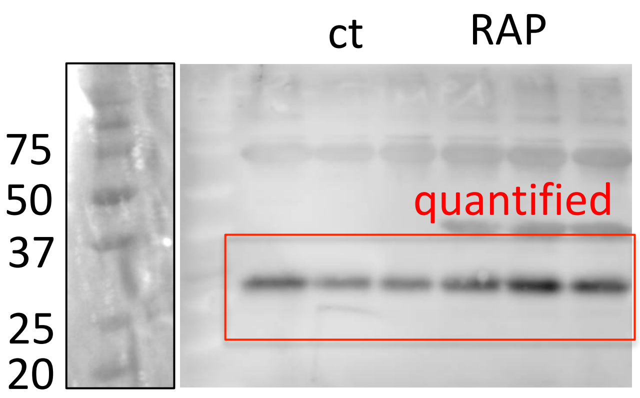

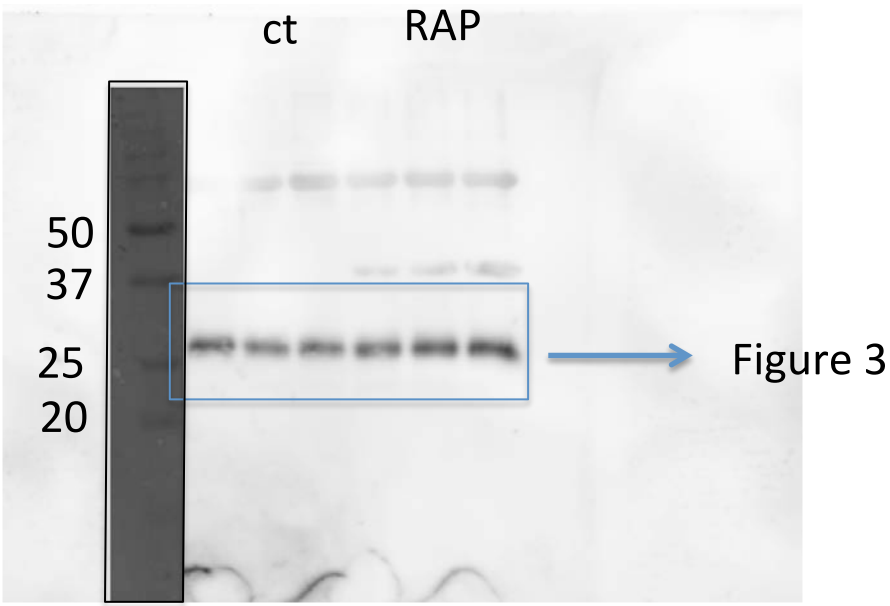

Blue square: bands in the MS. Red square + Blue square: bands quantified for statistics

TIMP2

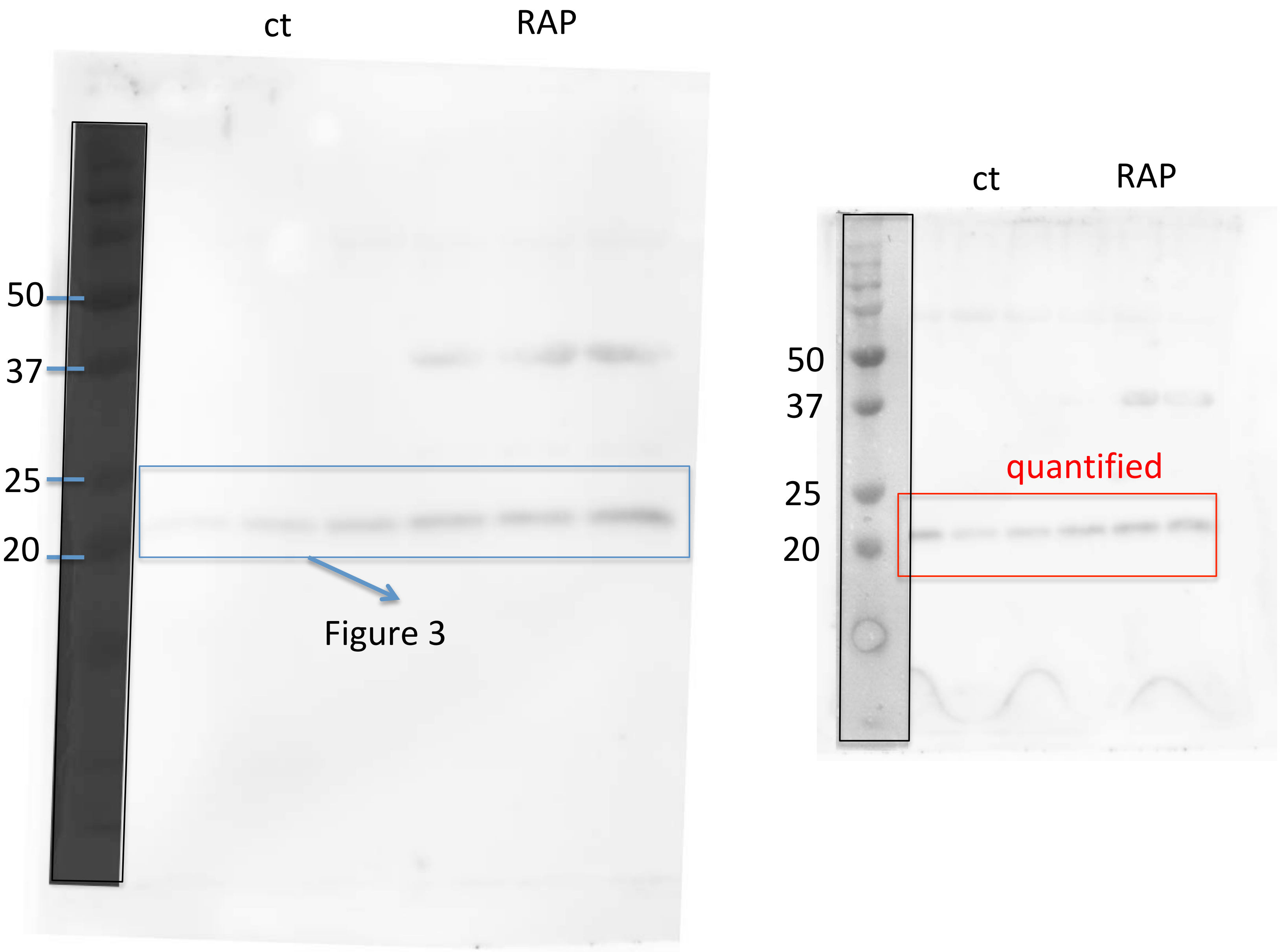

Blue square: bands in the MS. Red square + Blue square: bands quantified for statistics

SPARC

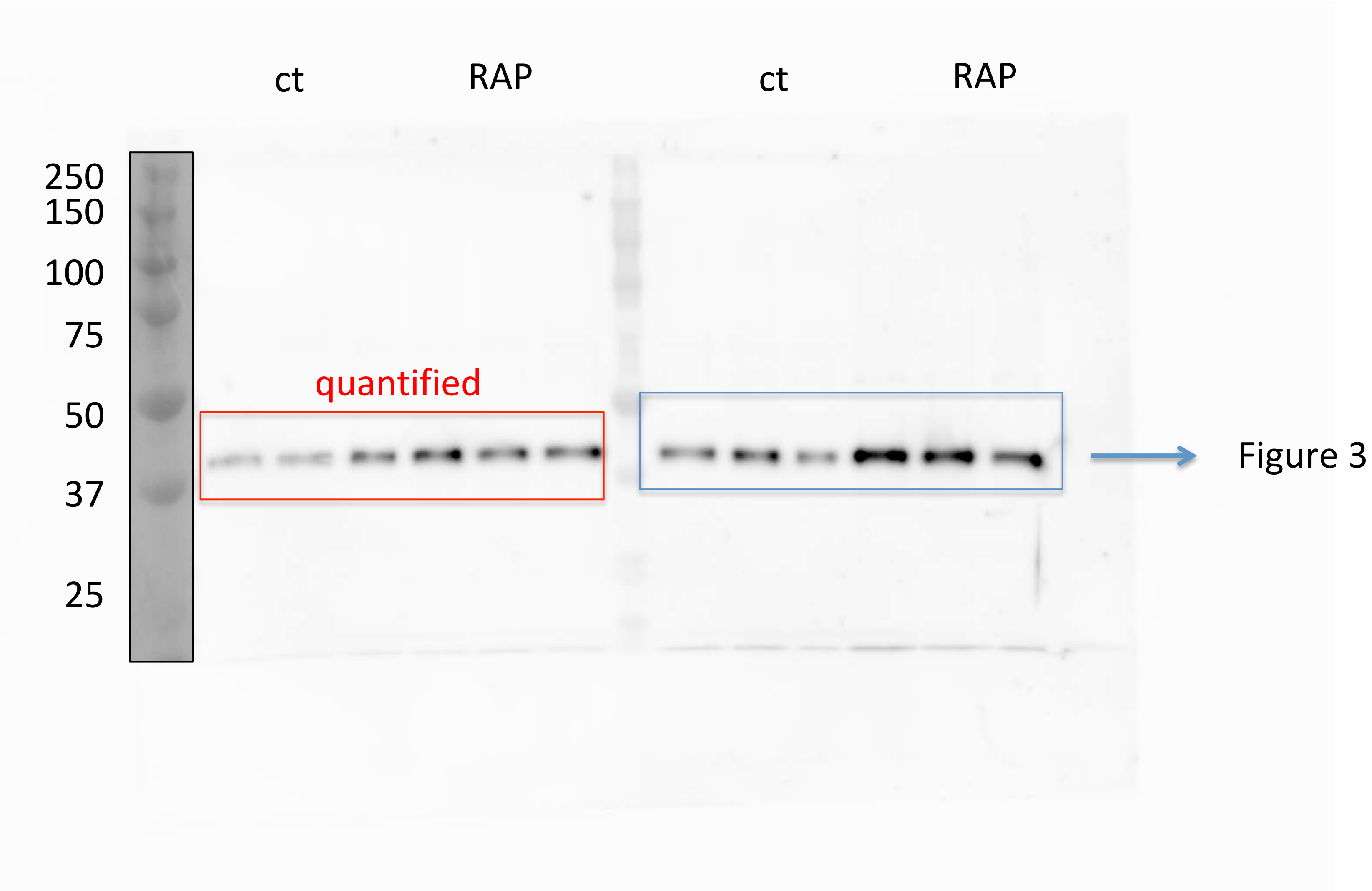

Blue square: bands in the MS. Red square + Blue square: bands quantified for statistics

MIF

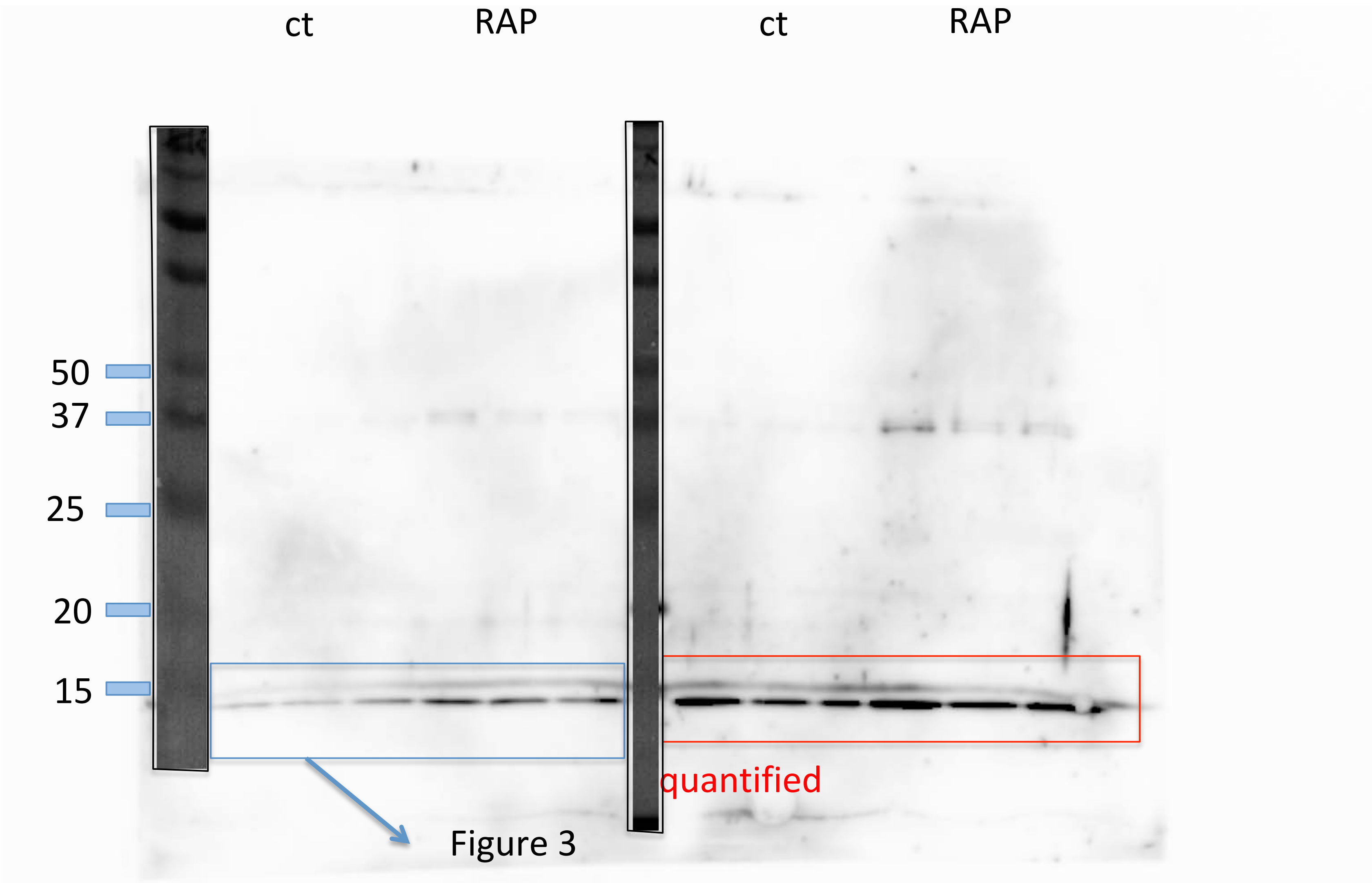

Blue square: bands in the MS. Red square + Blue square: bands quantified for statistics

Figure 5

sAPP

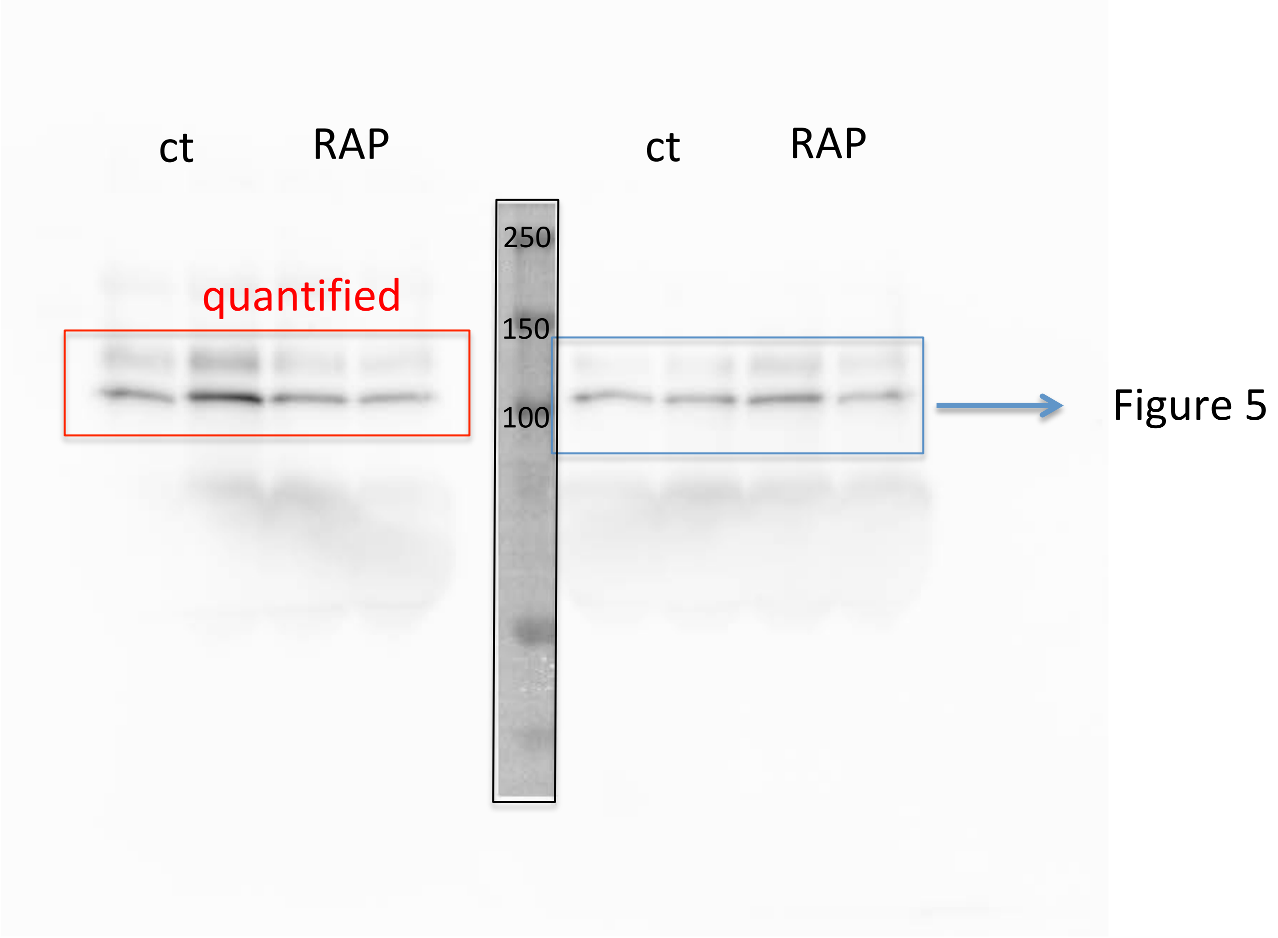

Blue square: bands in the MS. Red square + Blue square: bands quantified for statistics

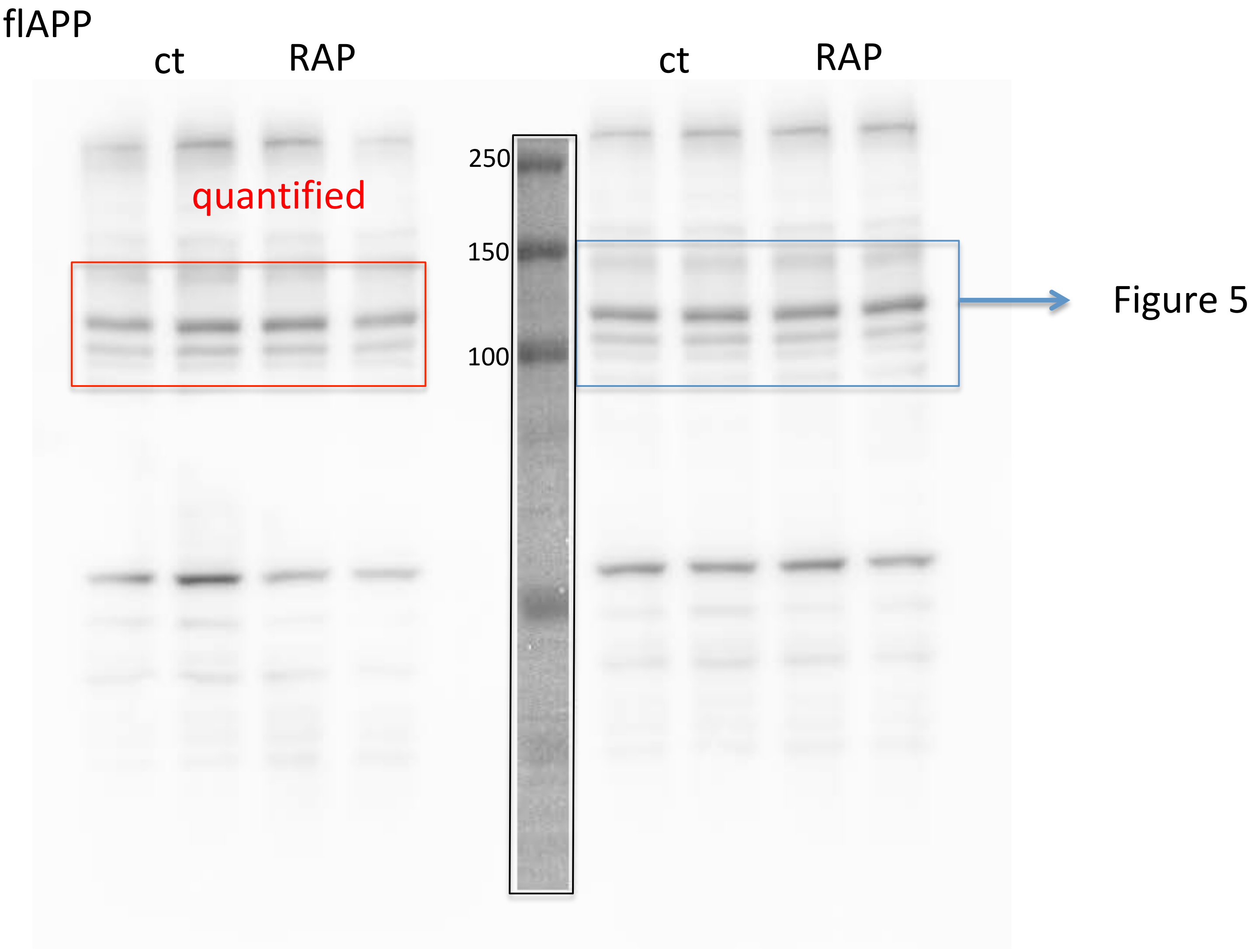

Blue square: bands in the MS. Red square + Blue square: bands quantified for statistics

actin

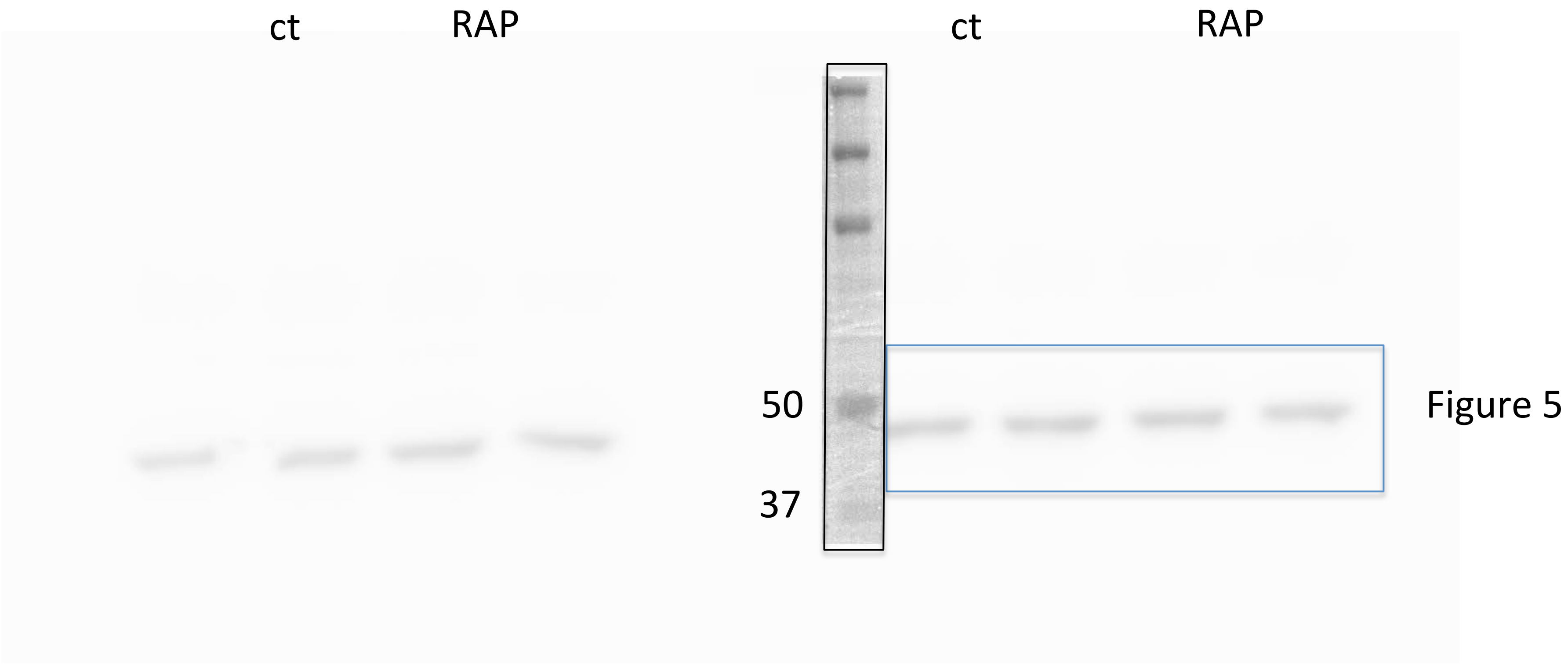

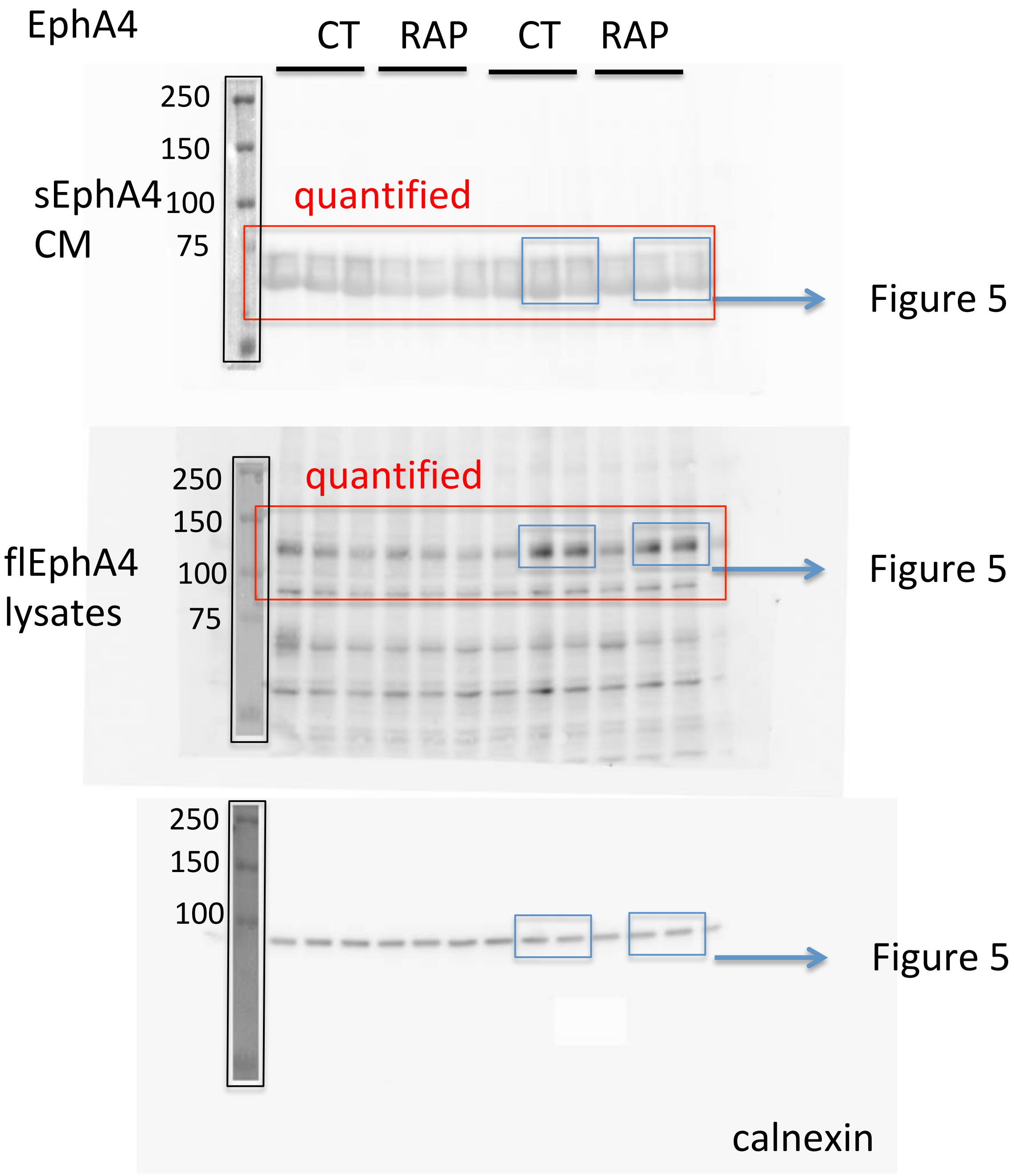

In the last version of the MS, the 2 bands that are not exactly next to each other, are clearly separated by a black line

Blue square: bands in the MS. Red square + Blue square: bands quantified for statistics
